# Lack of tumorigenesis and protumorigenic activity of human umbilical cord mesenchymal stem cells in NOD SCID mice

**DOI:** 10.1186/s12885-022-09431-5

**Published:** 2022-03-22

**Authors:** Jie He, Xiang Yao, Ping Mo, Kai Wang, Zai-ling Yang, Ni-ni Tian, Xiang-qing Zhu, Jing Zhao, Rong-qing Pang, Guang-ping Ruan, Xing-hua Pan

**Affiliations:** 1 Kunming Medical University, 920th Hospital Clinical College, Yunnan Province, Kunming, 650000 China; 2 Basic Medical Laboratory, 920th Hospital of Joint Logistics Support Force, PLA, Kunming, 650032 Yunnan Province China; 3 The Stem Cells and Immune Cells Biomedical Techniques Integrated Engineering Laboratory of State and Regions, Kunming, 650032 Yunnan Province China

**Keywords:** Tumorigenicity and promotion, Human umbilical cord mesenchymal stem cells, Injection, Tumor growth and metastasis

## Abstract

**Background:**

The tumorigenesis of infused umbilical cord mesenchymal stem cells (UC-MSCs) is being preclinically evaluated.

**Methods:**

We observed tumor formation in NOD SCID mice after a single subcutaneous injection of hUC-MSCs and the effect of these cells on tumor growth in tumor-bearing mice. Three generations (P5, P7, and P10) of hUC-MSCs (1 × 10^7^) from two donors (hUC-MSC1 and hUC-MSC2) were inoculated subcutaneously into NOD SCID mice. Subcutaneous transplantation models were established in NOD SCID mice with human cervical cancer HeLa cells (solid tumor) and human B cell lymphoma Raji cells (hematological tumor). Then, the animals were euthanized, gross dissection was performed, and tissues were collected. Various organs were observed microscopically to identify pathological changes and tumor metastasis.

**Results:**

In the tumorigenesis experiment, no general anatomical abnormalities were observed. In the tumor promotion experiment, some animals in the HeLa groups experienced tumor rupture, and one animal died in each of the low- and medium-dose hUC-MSC groups. The results may have occurred due to the longer feeding time, and the tumor may have caused spontaneous infection and death. Pathological examination revealed no metastasis to distant organs in any group. In the Raji tumor model, some animals in each group experienced tumor rupture, and one animal in the medium-dose hUC-MSC group died, perhaps due to increased tumor malignancy. Thus, hUC-MSCs neither promoted nor inhibited tumor growth. No cancer cell metastasis was observed in the heart, liver, spleen, lungs, kidneys or other important organs, except that pulmonary venule metastasis was observed in 1 animal in the model group.

**Conclusions:**

Injected hUC-MSCs were not tumorigenic and did not significantly promote or inhibit solid or hematological tumor growth or metastasis in NOD SCID mice.

**Supplementary Information:**

The online version contains supplementary material available at 10.1186/s12885-022-09431-5.

## Background

Umbilical cord mesenchymal stem cells (UC-MSCs) perform immunoregulatory functions and inhibit T cell proliferation and immune responses through cell–cell interactions and cytokine production. hUC-MSCs can inhibit the proliferation of mitogen-stimulated T lymphocytes, modulate T cell subsets to affect cytokine secretion, and participate in other mechanisms to exert immunomodulatory effects [1]. Studies have found that long-term in vitro-cultured bone marrow-derived mesenchymal stem cells (MSCs) can spontaneously transform and generate tumors [2]. Moreover, MSCs transfected with the telomerase reverse transcriptase gene (TERT) undergo transformation. Therefore, the tumorigenicity of UC-MSCs infused into patients has been a focus of preclinical evaluations.

Related studies have shown that the stability of MSCs in the tumor microenvironment is insufficient, and tumor growth may occur partly through the recruitment of peripheral stem cells and not only through the proliferation of the original tumor cells. Furthermore, MSCs have multidirectional differentiation potential and can differentiate into matrix components. In addition, chemokines and cytokines in the tumor microenvironment can induce the migration of MSCs to such microenvironments. This MSC migration promotes tumor stroma formation, which can lead to mutations in tumor cells and cancerous growth in the body [3, 4]. These reports all indicate that MSCs can promote tumor cell growth both in vivo and in vitro.

The hUC-MSCs used for injection are biological products developed by the National and Local Joint Engineering Laboratory of Stem Cell and Immune Cell Biomedicine Technology. These products are intended to be delivered via intravenous drip for the treatment of senile degenerative diseases. To evaluate the safety of this clinical intervention, tumor incidence in NOD SCID mice was monitored after the subcutaneous injection of hUC-MSCs to evaluate tumorigenicity, and the protumorigenic activity of hUC-MSCs was evaluated in subcutaneous models of a solid tumor (human cervical cancer HeLa cells) and hematological cancer (human B cell lymphoma Raji cells) in NOD SCID mice.

In this study, the tumorigenic and tumor-promoting effects of mesenchymal stem cells were studied from the perspective of a preclinical safety evaluation of a biological product. Research involving mesenchymal stem cells of different cell sources, different passages and different doses is relatively systematic and comprehensive, basically covering all possibilities of clinical use. This study is the most comprehensive evaluation of the safety of mesenchymal stem cell therapy reported to date. The results can be referenced, and the tumorigenic and tumor-promoting results in animals are more reliable than in vitro test results.

## Methods

### Materials

The hUC-MSCs used for injection were provided by the Stem Cells and Immune Cells Biomedical Techniques Integrated Engineering Laboratory of State and Regions at the 920th Hospital of Joint Logistics Support Force, PLA; human embryonic lung fibroblasts (MRC-5), human cervical cancer cells (HeLa), and human B cell lymphoma cells (Raji) were purchased from the Cell Center of the Institute of Basic Research, Chinese Academy of Sciences. We obtained these cell lines from the Cell Bank within 6 months. Reauthentication (STR analysis) of cell lines (hUC-MSC1, hUC-MSC2, MRC-5, HeLa, and Raji cells) is required for serially passaged cells used for more than 6 months after receipt from an internationally recognized cell bank. All the experiments were performed with mycoplasma-free cells; mycoplasma screening was performed by PCR, and these results are included in the responses to the reviewers.

SPF-grade NOD SCID mice were purchased from Beijing Weitong Lihua Laboratory Animal Technology Co., Ltd. under certificate numbers 0310377, 0,301,028, and 0,308,898. The animals were purchased at 4–6 weeks of age with a weight range of 18–23 g, and they were housed in an SPF animal room.

### Methods

#### Identification of Surface Markers Expressed by UC-MSCs and Preparation for Injection

UC-MSCs were collected in the logarithmic growth phase, washed 3 times with physiological saline, divided into tubes (1 × 10^6^ cells per tube), and incubated with 10 μl CD105-PE, CD73-FITC, CD90-PerCP-Cy5.5, CD34-PE, CD45-FITC or isotype control at 4 °C in the dark for 30 min. After washing with phosphate buffer to remove the unbound antibodies, the expression level of the surface markers was detected with a flow cytometer.

In the tumorigenesis experiments, UC-MSCs were collected, centrifuged, counted, and then adjusted to 5 × 10^7^ cells/ml with serum-free DMEM/F12 culture medium for later use.

In the tumor promotion experiment, UC-MSC suspensions of various concentrations were prepared so that the suspension contained 1% human albumin, 1050 IU/4 × 10^7^ cells low-molecular-weight heparin calcium, and 2% DMSO. At cell viabilities of 80% ~ 100%, the cell concentration was 80% ~ 120% of the labeled cell concentration. The prepared cell suspension was stored or transported in an ice box.

#### Tumorigenesis experiment

NOD SCID mice were randomized into 8 groups with 10 mice each, and each group included equal numbers of male and female mice. For the experimental groups, the cells were derived from three generations (P5, P7, and P10) or two batches of cells (hUC-MSC1 and hUC-MSC2); HeLa cells served as the positive control, and MRC-5 cells served as the negative control. The positive control group was injected with 1 × 10^6^ cells/mouse, and the other groups received 1 × 10^7^ cells/mouse. The mice underwent 16 weeks of continuous observation after subcutaneous inoculation into the right forelimb axillary. Mouse body weight was measured twice a week before and after cell inoculation, and the nodule volume was continuously observed and measured after cell inoculation. During the experiment, the nodules had a tendency to diminish, so half of the animals with nodules were euthanized before the nodules completely disappeared, and the other half were subjected to continued observation until the nodules disappeared completely and then until the end of the 16th week of the experiment, at which point the remaining animals were euthanized. For animals that did not develop nodules, half were euthanized at day 21 (D21) after inoculation, and the other half underwent continued observation until the end of the 16th week. All the euthanized animals were subjected to gross dissection, and the nodules or tissues at the inoculation site were harvested for histopathological examination.

Thirty-two animals were selected and randomly divided into 4 groups with 8 animals each: the negative control group, low-dose hUC-MSC group (1 × 10^7^ cells/kg), medium-dose hUC-MSC group (2 × 10^7^ cells/kg), and high-dose hUC-MSC group (4 × 10^7^ cells/kg). hUC-MSCs were injected via tail vein. The cells were administered by tail vein injection once each in weeks 1, 3, and 5, for a total of 3 injections. At the end of the experiment, gross dissection of all animals was carried out; the main organs, including the heart, liver, spleen, lungs, kidneys, and brain, were examined for whether there is tumor formation. The tumorigenesis potential of MSCs administered via tail vein injection was evaluated.

#### Tumor promotion experiment

HeLa and Raji cells (0.2 ml, 5 × 10^7^ cells/ml) were injected subcutaneously into NOD SCID mice to establish a xenograft tumor model, and tumor-bearing animals that exhibited vigorous growth, no ulceration, and good health were selected. Tumors were collected under aseptic conditions, and tumor masses of 1.5–3 mm^3^ were subcutaneously inoculated into the right axillary region of NOD SCID mice. After inoculation, tumor growth was monitored. When the average tumor volume reached 50–100 mm^3^, the tumor size was monitored. Animals with tumor volumes that were too large and those without tumors were not selected for further experiments.

Thirty-two model animals with each tumor cell line were selected and randomly divided into 4 groups with 8 animals each: the model group, low-dose hUC-MSC group (1 × 10^7^ cells/kg), medium-dose hUC-MSC group (2 × 10^7^ cells/kg), and high-dose hUC-MSC group (4 × 10^7^ cells/kg). The cells were administered by tail vein injection once each in weeks 1, 3, and 5, for a total of 3 injections. The HeLa cell groups were observed for 56 days after the first injection, and the Raji cell groups were observed for 37 days. General physiological indicators, including the animal's mental state, behavior, and food intake, were observed every day. The long diameter, short diameter and weight of the tumor were measured and recorded twice a week, the tumor volume was calculated, and the tumor growth curves were compared between the groups. The relative tumor volume (RTV) was calculated as RTV = Vt/V0, where Vt is the current tumor volume, and VO is the initial tumor volume. The relative tumor proliferation rate (T/C%) was calculated as T/C% = average RTV of the treatment group/average RTV of the control group × 100%. T/C% ≤ 40% with P < 0.05 indicated statistically significant tumor growth inhibition, T/C% ≥ 140% indicated increased tumor growth, and 40% < T/C% < 140% indicated neither promoted nor inhibited tumor growth. After observation, the animals were anaesthetized with pentobarbital sodium, blood was collected, and the animals were euthanized. The tumor nodules were dissected and weighed. The tumor weight of each group was measured to calculate the tumor inhibition rate IRTW (%) = (W model group-W treatment group)/W model group × 100%. At the end of the experiment, gross dissection of all animals was carried out; the main organs, including the heart, liver, spleen, lungs, kidneys, and brain, were examined for metastasis, and histopathological examination of the abovementioned tissues and tumor nodules was conducted.

#### Statistical analysis

SPSS 26.0 statistical software was used to perform statistical analysis on the weight, nodule volume, organ weight, and organ coefficient of different groups of animals. The data are expressed as the mean ± standard deviation (x ± s) For nodule volume, only the average of each group is presented, and no statistical analysis was required. Specific analysis was carried out according to the following procedure: Levene’s test was used to test the homogeneity of variance. If there was no statistical significance (*P* > 0.05), one-way analysis of variance (ANOVA) was used for statistical analysis. If ANOVA suggested statistical significance (*P* ≤ 0.05), Dunnett’s test (parametric method) was used for comparative analysis. If the variance was not uniform (*P* ≤ 0.05), the Kruskal–Wallis test was used. If the Kruskal–Wallis test suggested statistical significance (*P* ≤ 0.05), then the Mann–Whitney method was used for pairwise comparisons between means.

## Results

### Identification of surface markers of UC-MSCs

Antibody-labeled cells were shown to have high expression of CD105, CD73, and CD90 and low or no expression of CD45 and CD34 by flow cytometry. These results met the criteria for identifying mesenchymal stem cells by surface marker expression (see Supplementary Figure S1).

### Results of the tumorigenicity experiment

#### General clinical observation

During the observation period, all the mice were in a good mental health and exhibited normal behaviors. The animal weights showed continuous growth. There was no significant difference in the animal weight between the groups (*P* > 0.05). On D55, tumors began to appear in 6 animals in the positive control group. Ulceration gradually worsened, and all the animals in this group were euthanized on D61. The animal weight data are shown in Table S1 of the Supplementary Materials.

#### Clinical and histological observations of subcutaneously injected nodules

**Fig. 1:**
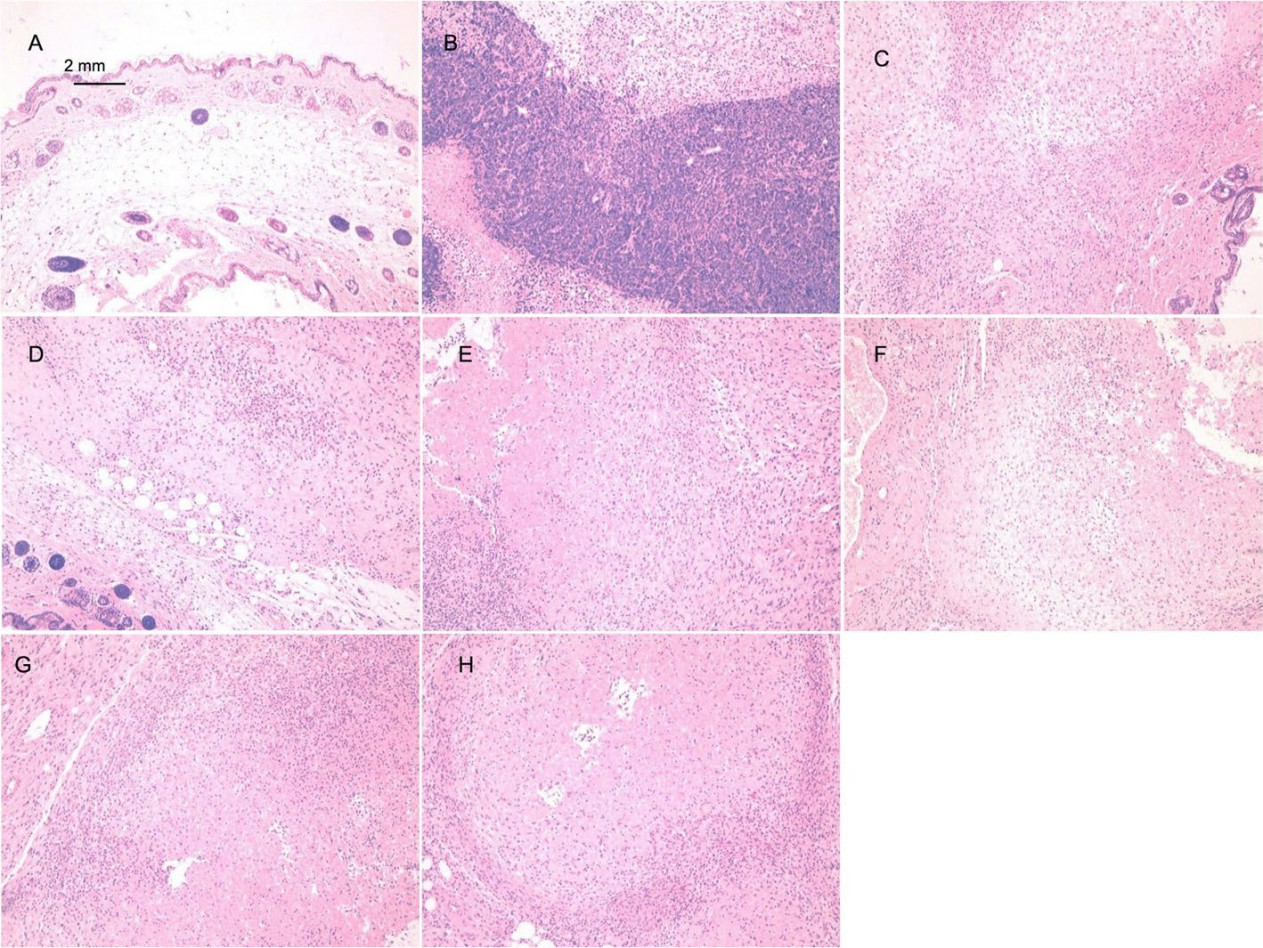
Histopathological examination of the inoculation site of NOD SCID mice injected with hUC-MSCs (HE staining, 10 ×). **A** Animals in Group 1 were euthanized, and no obvious abnormalities were seen at the inoculation site. **B** Animals in Group 2 were euthanized, and tumor nodules with central necrosis were observed at the inoculation site. **C–H** Animals in Groups 3, 4, 5, 6, 7, and 8 were euthanized. The stem cell mass at the inoculation site was necrotic, and the surrounding tissues exhibited fibroblastic proliferation accompanied by lymphocyte infiltration

Negative control group (group 1): After cell inoculation, no obvious nodules were seen at the injection site. Half of the animals were euthanized 21 days after inoculation, and no abnormalities in various organs or tissues were observed during the gross autopsy. Microscopic examination revealed that injected cells remained in one animal; there were 2 lymphocyte nodules at the injection site, accompanied by fibroblast proliferation nodules. The remaining half of the animals were observed until the end of the 16th week, at which point they were euthanized. There was no nodule growth at the inoculation site or in the surrounding tissues of these animals. The nodule volume data are shown in Table S2 of the Supplementary Materials. The microscopic examination showed no abnormalities (Fig. 1A).

Positive control group (group 2): Starting on D8 after cell inoculation, nodules gradually appeared at the inoculation site and increased in size. By D61, most animals (7/10) had nodules over 20 mm in diameter, which were classified as tumors. All the animals in this group developed tumors and ulceration. Considering animal welfare, all the animals in this group were euthanized. The gross autopsy showed nodules on the right side of the armpit in all 10 animals, but no abnormalities in organ structure were observed. The microscopic examination of axillary nodules revealed these to all be tumor nodules. For the statistical analysis of the nodules, see Table S2 of the Supplementary Materials. No abnormalities were detected in the microscopic examination (Fig. 1B).

**Fig. 2:**
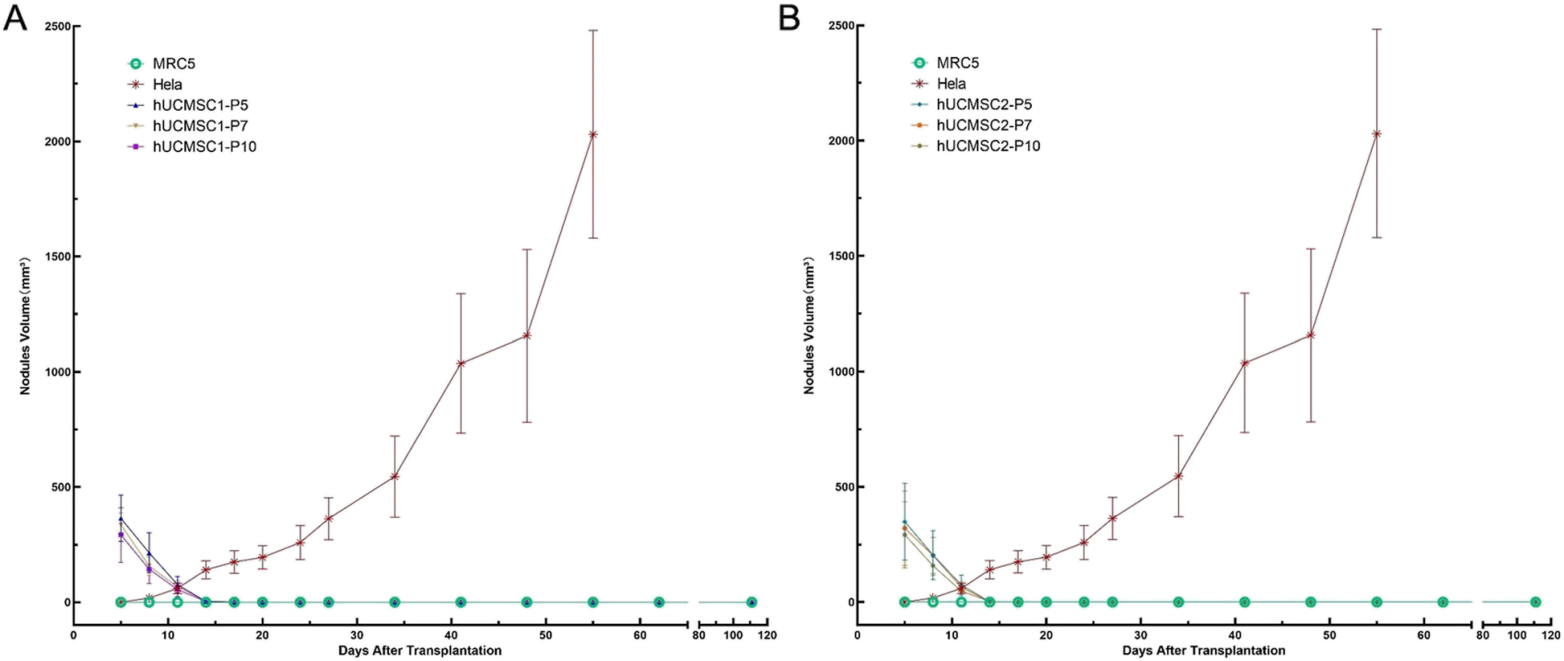
Changes in nodule growth after hUC-MSC inoculation. **A** Graph of nodule growth in the hUC-MSC1 group after treatment. Compared with the negative control group, the hUC-MSC1-P5, hUC-MSC1-P7, and hUC-MSC1-P10 groups showed no significant difference in nodule growth. **B** Graph of nodule growth in the hUC-MSC2 group after treatment. Compared with the negative control group, the hUC-MSC2-P5, hUC-MSC2-P7, and hUC-MSC2-P10 groups showed no significant difference in nodule growth

hUC-MSC1 groups (group 3: P5, group 4: P7, and group 5: P10): No obvious nodules were observed at the injection site after cell inoculation. At D5, all 10 animals in each group had nodules at the inoculation site. These nodules gradually decreased in size, and half of the animals (5/10) were euthanized before the nodules disappeared on D11. Gross examination revealed no abnormalities in various organs or tissues. Microscopic examination of the hUC-MSC1-P7 group revealed 1 animal with local fibrous tissue proliferation accompanied by lymphocyte infiltration, but the animals showed no abnormalities. The remaining half of the animals continued to be observed; all the nodules disappeared by D17, and observation continued until the end of 16 weeks, when the animals were euthanized. During this period, no nodules were seen at the inoculation site or the surrounding area. The statistical analysis of nodules is shown in Supplementary Material Table S2. The nodule growth trends in the hUC-MSC1 groups and the positive control group are shown in Fig. 2A. Gross anatomical observation showed no nodules at the inoculation site and no abnormalities in various organs or tissues; moreover, microscopic evaluations revealed no local abnormalities at the inoculation site (Fig. 1C, D, E).

hUC-MSC2 groups (group 6: P5, group 7: P7, and group 8: P10): No obvious nodules were seen at the injection site after cell inoculation. At D5, all 10 animals in each group had nodules at the inoculation site; these nodules gradually decreased in size, and on D11, half of the animals (5/10) were euthanized before the nodules disappeared. There were no gross anatomical abnormalities in organs or tissues, and local stem cell clusters were observed by microscopy in only 2/5 animals. These 2 mice also showed mass necrosis and the proliferation of surrounding fibrous tissue, which was accompanied by lymphocyte infiltration; however, no abnormalities were observed in the remaining animals. Observation continued for the remaining animals in each group. By D17, all the nodules disappeared. Observation continued until the end of the 16th week, at which point the animals were euthanized. During this period, no nodules were seen at the inoculation site or elsewhere. The nodule volume data are shown in Table S2 of the Supplementary Materials. Nodule growth in the hUC-MSC2 groups and the positive control group is shown in Fig. 2B. Gross anatomical observation showed no nodules at the inoculation site and no abnormalities in various organs or tissues, and microscopic analysis revealed no local abnormalities at the inoculation site (Fig. 1F, G, H).

#### Intravenous injection tumorigenic test results

The anatomical and pathological examination results of the whole body of 32 animals are shown in Table 1. There was no tumor formation in the heart, liver, spleen, lungs, kidneys, and brain and other major organs. Conclusion: hUC-MSCs were transplanted intravenously had no tumor formation in the low, medium and high dose groups. It indicated that hUC-MSCs are safe by intravenous infusion.

**Table 1:** Intravenous injection tumorigenic test results

Dose	heart	liver	spleen	lungs	kidneys	brain
1 × 10^7^ cells/kg	No tumor seen	No tumor seen	No tumor seen	No tumor seen	No tumor seen	No tumor seen
2 × 10^7^ cells/kg	No tumor seen	No tumor seen	No tumor seen	No tumor seen	No tumor seen	No tumor seen
4 × 10^7^ cells/kg	No tumor seen	No tumor seen	No tumor seen	No tumor seen	No tumor seen	No tumor seen

### Results of the tumor promotion experiment

#### Effect on HeLa cell tumor growth

Most of the animals were in good mental condition, with little changes in body weight. There were no significant differences in body weight between each experimental group and the model group (*P* > 0.05). No animals died in the model group, but tumor rupture occurred in 4 animals on D39.

Low-dose hUC-MSC group (1 × 10^7^ cells/kg): One animal died suddenly on D44, and no obvious gross anatomical abnormalities were observed. On D29, 4 animals successively developed tumor ulceration.

Medium-dose hUC-MSC group (2 × 10^7^ cells/kg): One animal died suddenly on D29 with no obvious gross abnormalities. On D18, 3 animals successively developed tumor ulceration.

High-dose hUC-MSC group (4 × 10^7^ cells/kg): No animals in this group died, but tumors ruptured in 2 animals on D43.

For weight data, see Table S3 of the Supplementary Materials.

After the first experimental cell injection, the long and short tumor diameters were measured twice a week, and the tumor volume (V) and RTV (Vt/V0) were calculated. The following results were recorded.

Model group: The tumor nodules grew steadily. The average tumor volume at D57 (euthanasia) was 3028.58 mm^3^, yielding an RTV of 40.26 ± 7.01.

Low-dose hUC-MSC group (1 × 10^7^ cells/kg): Tumor nodules grew steadily, and the average tumor volume on D57 was 2839.68 mm^3^, yielding an RTV of 43.17 ± 15.82. This group showed no significant difference compared with the model group (*P* > 0.05).

Medium-dose hUC-MSC group (2 × 10^7^ cells/kg): The tumor nodules grew steadily, and the average tumor volume at D57 was 2316.22 mm^3^, which was 31.44 times larger than the initial tumor volume (RTV: 31.44 ± 7.47). Compared with the model group, this group showed no significant difference (*P* > 0.05).

High-dose hUC-MSC group (4 × 10^7^ cells/kg): The tumor nodules grew steadily, and the average tumor volume on D57 was 2625.85 mm^3^, which was 43.17 times larger than the initial tumor volume (RTV: 35.99 ± 9.38). The difference between this group and the model group was not significant (*P* > 0.05). There was no significant difference in average tumor volume at each time point between the high-dose and low-dose groups (*P* > 0.05), indicating no significant dose-dependent effects in these experiments.

**Fig. 3:**
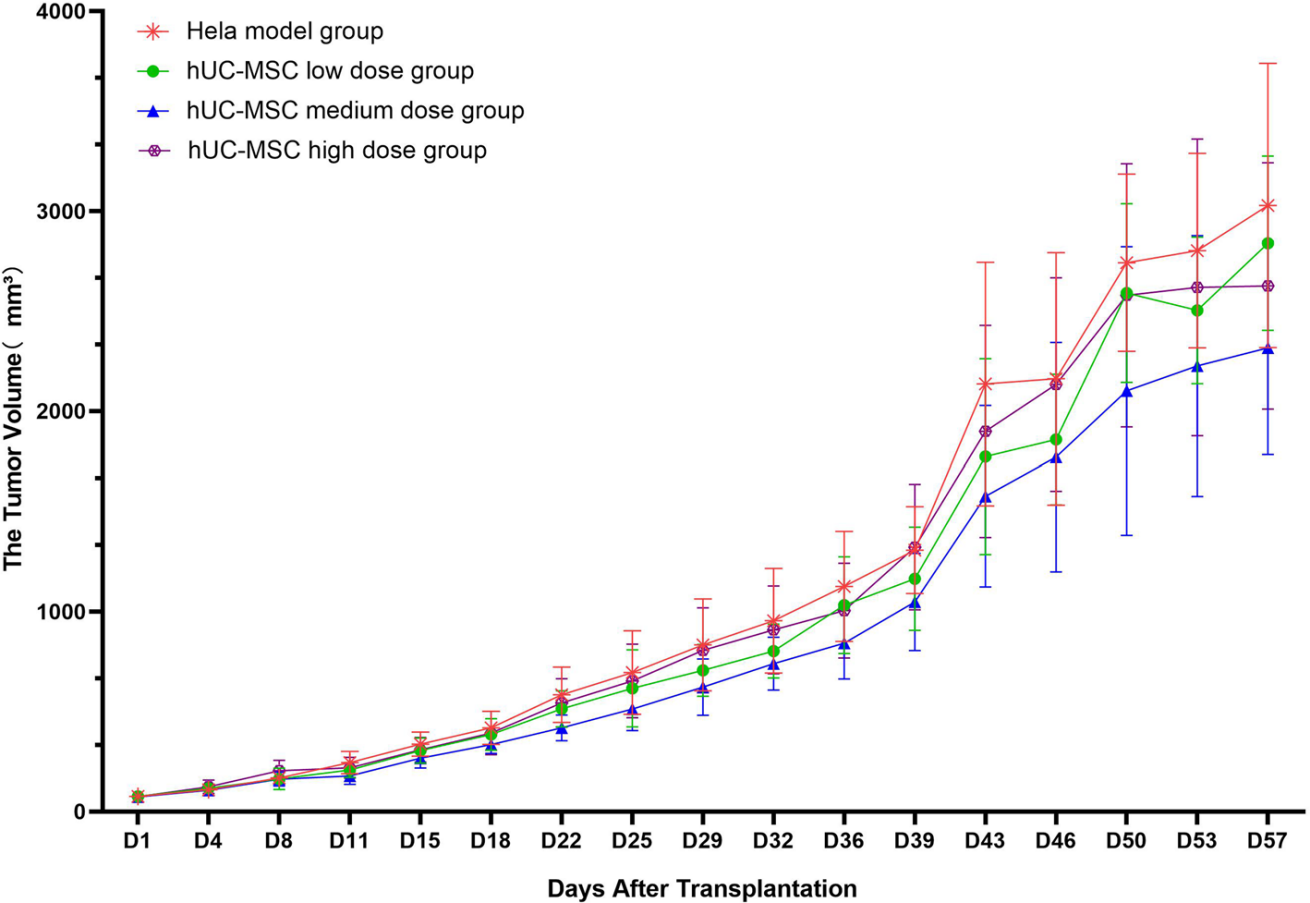
Changes in tumor volume in each HeLa group. There were no significant differences between the low-, medium- and high-dose hUC-MSC groups and the model group. This result indicated that low, medium and high doses of hUC-MSCs did not significantly promote or inhibit the growth and metastasis of HeLa cell-derived tumors

The tumor volume data are shown in Table S4 of the Supplementary Materials, and the growth trend is shown in Fig. 3.

The relative tumor proliferation rate (T/C%) was determined by comparing the average RTV of each group with that of the model group. Tumor growth inhibition was indicated by T/C% ≤ 40% and *P* < 0.05, whereas T/C% ≥ 140% indicated tumor growth promotion; results of 40% < T/C% < 140% indicated neither an oncogenic nor inhibitory effect.

The results were as follows:

Low-dose hUC-MSC group (1 × 10^7^ cells/kg): T/C% reached the maximum value of 110.69% on D4 and the minimum value of 91.12% on D11; the value on D57 was 107.23%.

Medium-dose hUC-MSC group (2 × 10^7^ cells/kg): T/C% reached the maximum value of 97.99% on D4 and the minimum value of 74.69% on D11; the value on D57 was 78.11%.

High-dose hUC-MSC group (4 × 10^7^ cells/kg): T/C% reached the maximum value of 120.30% on D8 and the minimum value of 89.39% on D57.

The results showed that the T/C5 values for the hUC-MSC treatment groups were 40% < T/C% < 140% during the entire experiment, indicating that hUC-MSCs were not oncogenic or tumor suppressive in animals injected with 1 × 10^7^, 2 × 10^7^, or 4 × 10^7^ HeLa cells/kg. Tumor-bearing mice showed neither an increase nor a decrease in tumor growth.

The RTV and T/C% data are shown in Tables S5 and S6 of the Supplementary Materials.

At the end of the experiment (D57), the animals were euthanized, the tumors were dissected and weighed, and the average tumor weight in each group was calculated and compared.

The average tumor weights in the model, low-dose hUC-MSC (1 × 10^7^ cells/kg), medium-dose hUC-MSC (2 × 10^7^ cells/kg), and high-dose hUC-MSC groups (4 × 10^7^ cells/kg) were 2.455 g, 2.349 g, 2.082 g, and 2.491 g, respectively; there were no significant differences in average tumor weight between the model group and the hUC-MSC groups (*P* > 0.05).

The IRTW was calculated based on the average tumor weight in each group; the values were 4.30%, 15.19%, and -1.45% in the low-dose (1 × 10^7^ cells/kg), medium-dose (2 × 10^7^ cells/kg), and high-dose hUC-MSC groups (4 × 10^7^ cells/kg), respectively.

The tumor weight data are shown in Table S7 of the Supplementary Materials.

Gross anatomical observations revealed no abnormalities in the animals that experienced sudden death (one in the low-dose group and one in the middle-dose group). Inoculated tumor nodules with a diameter of approximately 2 cm were observed in the armpits of animals scheduled for euthanization, and no obvious abnormalities were observed in the remaining animals.

Among the animals that died in the low-dose group, there was moderate (+ +) neutrophil and monocyte infiltration in the epicardium, moderate liver congestion, extensive (+ +  + +) mononucleosis in the spleen, extensive necrosis of renal tubules, and tumor nodules in the axilla. Most of the tissue sections were necrotic.

In the dead animals in the middle-dose group, necrosis was primarily observed in the center of axillary tumor nodules.

**Fig. 4:**
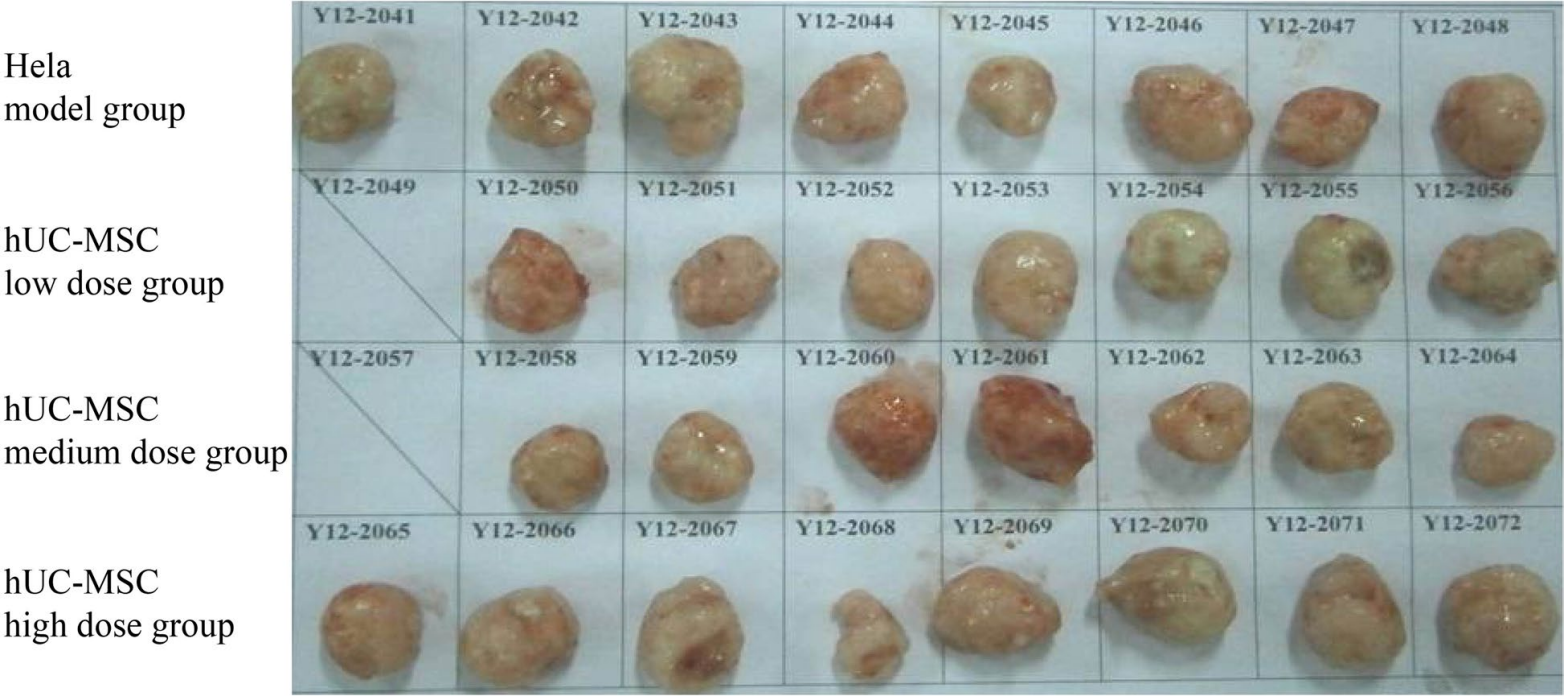
Photos of tumors from each group on day 57 (HeLa). The tumors in each group were the same size, and no differences were significant. This result indicated that low, medium and high doses of hUC-MSCs did not significantly promote or inhibit the growth and metastasis of HeLa cell-derived tumors

**Fig. 5:**
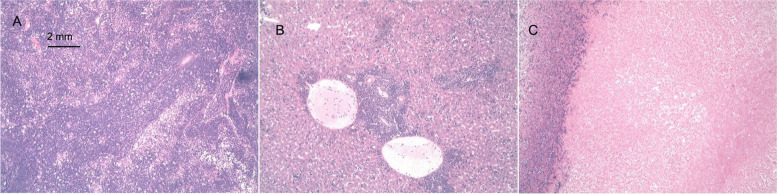
Histopathological examination of tissues and HeLa cell-derived tumors after the injection of hUC-MSCs into NOD SCID mice (HE staining, 10 ×). **A** The lymphocytes in the spleens of the animals in the low-dose group exhibited proliferation and a dense arrangement. **B** In the medium-dose group, more monocytes accumulated near the central vein in the liver. **C** The tumor cell masses in the axilla of the model group contained large areas of central necrosis

The histopathological changes in the organ tissues of the animals that died in the low-dose group suggested severe systemic infection, acute inflammation of the epicardium, enhanced spleen function, and increased mononuclear cell numbers; liver congestion was caused by the death of animals without bleeding, and due to the severe infection, bacterial toxins caused extensive renal tubule necrosis, resulting in kidney failure and death. The central area of ​​the axillary tumor was necrotic due to ischemia and hypoxic due to rapid growth. Additionally, the dead animal in the medium-dose group exhibited no abnormal changes in organs, and the cause of death was unknown. The general anatomical and pathological results are shown in Figs. 4 and 5.

#### Effect on Raji cell tumor growth

Most of the animals were in good mental condition, with little change in body weight, and there were no significant differences between the treatment groups and the model group (*P* > 0.05).

Model group: No animals died, but 4 animals experienced tumor rupture starting on D32.

Low-dose hUC-MSC group (1 × 10^7^ cells/kg): No animals died, tumor rupture occurred in 5 animals starting on D29, and one animal exhibited hindlimb weakness.

Medium-dose hUC-MSC group (2 × 10^7^ cells/kg): One animal died suddenly on D34 with no obvious gross abnormality. Starting on D29, all the remaining 7 animals experienced tumor rupture.

High-dose hUC-MSC group (4 × 10^7^ cells/kg): No animals died. On D29, 6 animals developed tumor ulcers, and one of them exhibited hindlimb weakness.

For weight statistics, see Table S8 of the Supplementary Materials.

Model group: The tumor nodules grew steadily. The average tumor volume was 2474.87 mm^3^ on D36 (euthanasia), yielding an RTV of 41.68 ± 18.20.

Low-dose hUC-MSC group (1 × 10^7^ cells/kg): The tumor nodules grew steadily, and the average tumor volume on D57 (euthanasia) was 2878.42 mm^3^, which was 47.69 times larger than the initial tumor volume (RTV: 47.69 ± 15.55). Compared with the model group, this group showed a significant increase in average tumor volume at D32 (*P* ≤ 0.05).

Medium-dose hUC-MSC group (2 × 10^7^ cells/kg): The tumor nodules steadily increased in size, and the average tumor volume was 2813.67 mm^3^ at D36 (euthanasia), yielding an RTV of 53.60 ± 29.79. The difference in average tumor volume between this group and the model group was not significant (*P* > 0.05).

High-dose hUC-MSC group (4 × 10^7^ cells/kg): The tumor nodules grew steadily. By D36 (euthanasia), the average tumor volume was 2990.84 mm^3^, which was 48.26 times larger than the initial tumor volume (RTV: 48.26 ± 11.95). Compared with the model group, this group showed a significant increase in average tumor volume on D32 *(P* ≤ 0.05). There was no significant difference in average tumor volume at each time point between the high-dose and low-dose groups (*P* > 0.05), indicating no significant dose-dependent effects in this experiment.

**Fig. 6:**
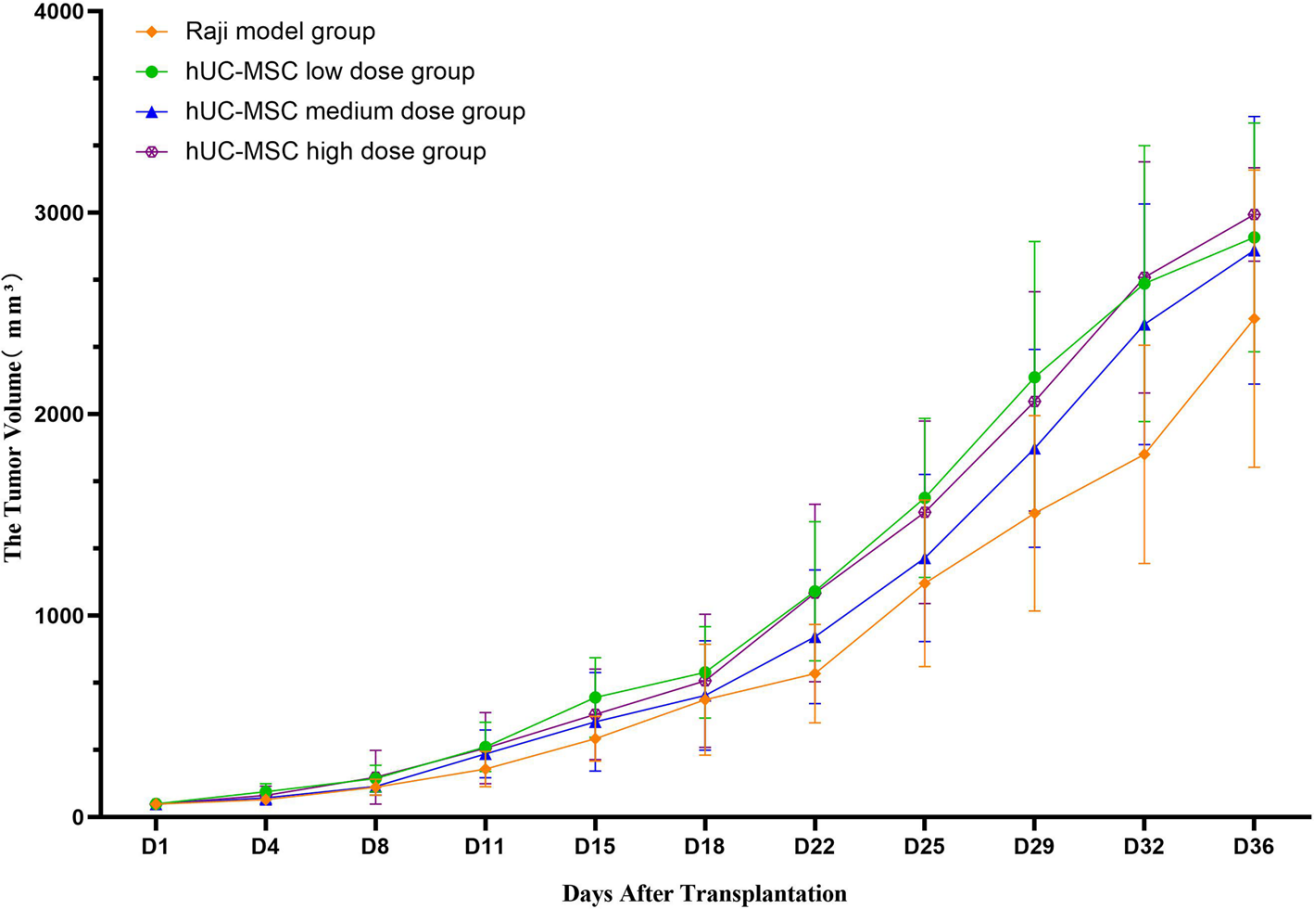
Changes in tumor volume in each Raji group. There were no significant differences between the low-, medium- and high-dose hUC-MSC groups and the model group. This result indicated that low, medium and high doses of hUC-MSCs did not significantly promote or inhibit the growth and metastasis of Raji cell-derived tumors

The tumor volume data are shown in Table S9 of the Supplementary Materials, and the growth trend is shown in Fig. 6.

The relative tumor proliferation rate (T/C%) was determined by comparing the average RTV of each group with that of the model group. The results of T/C% ≤ 40% and *P* < 0.05 indicated an inhibitory effect on tumor growth, whereas T/C% ≥ 140% indicated a protumorigenic effect; 40% < T/C% < 140% indicated no effect on tumor growth.

The results were as follows:

Low-dose hUC-MSC group (1 × 10^7^ cells/kg): T/C% reached the maximum value of 152.46% on D22 and the minimum value of 114.42% on D36. The only significant difference compared with the model group was the higher average RTV on D4 (*P* ≤ 0.01).

Medium-dose hUC-MSC group (2 × 10^7^ cells/kg): T/C% reached the maximum value of 144.01% on D32 and the minimum value of 99.06% on D18; T/C% was 128.60% on D36.

High-dose hUC-MSC group (4 × 10^7^ cells/kg): T/C% reached the maximum value of 150.68% on D22 and the minimum value of 113.13% on D18; T/C% on D36 was 115.79%.

The results showed T/C% > 140 for hUC-MSCs in each group at some time points, but the differences in RTV between the experimental and model groups were not significant, indicating that 1 × 10^7^, 2 × 10^7^, and 4 × 10^7^ hUC-MSCs/kg may have a certain ability to promote tumor growth in Raji cell tumor-bearing mice.

The RTV and T/C% data are shown in Tables S10 and S11 of the Supplementary Materials.

At the end of the experiment (D36), the animals were euthanized, the tumors were harvested and weighed, and the average tumor weight in each group was calculated and compared.

The average tumor weights in the model, low-dose hUC-MSC (1 × 10^7^ cells/kg), medium-dose hUC-MSC (2 × 10^7^ cells/kg), and high-dose hUC-MSC groups (4 × 10^7^ cells/kg) were 2.214 g, 2.509 g, 2.607 g, and 2.796 g, respectively; compared with the model group, the treatment groups showed no significant difference in average tumor weight (*P* > 0.05).

The IRTW was calculated based on the average tumor weight in each group, and the values were -13.34%, -17.76%, and -26.31% in the low-dose (1 × 10^7^ cells/kg), medium-dose (2 × 10^7^ cells/kg), and high-dose hUC-MSC groups (4 × 10^7^ cells/kg), respectively.

The tumor weight data are shown in Table S12 of the Supplementary Materials.

In the gross anatomical evaluation, an inoculated tumor nodule with a diameter of approximately 2 cm was observed in the armpit of an animal that died suddenly, but no anatomical abnormalities were observed. Inoculated tumor nodules of approximately 2 cm in diameter were observed under the armpits of the animals in each group, with no accompanying abnormalities.

Except for a metastatic cancer cell mass in the pulmonary venules of 1 animal in the model group, no cancer cell metastasis was detected in the heart, liver, spleen, lungs, kidneys or other important organs of the animals in the other groups. Some animals showed splenic extramedullary hematopoiesis, but no dose–effect relationship was identified, indicating that this finding represented a spontaneous physiological response of the rodents. All the central areas of the axillary inoculation tumors showed different degrees of necrosis. Some spontaneous diseases that are common in animals, such as interstitial pneumonia, lung tissue focal bleeding, and extramedullary hematopoiesis in the spleen, were observed.

**Fig. 7:**
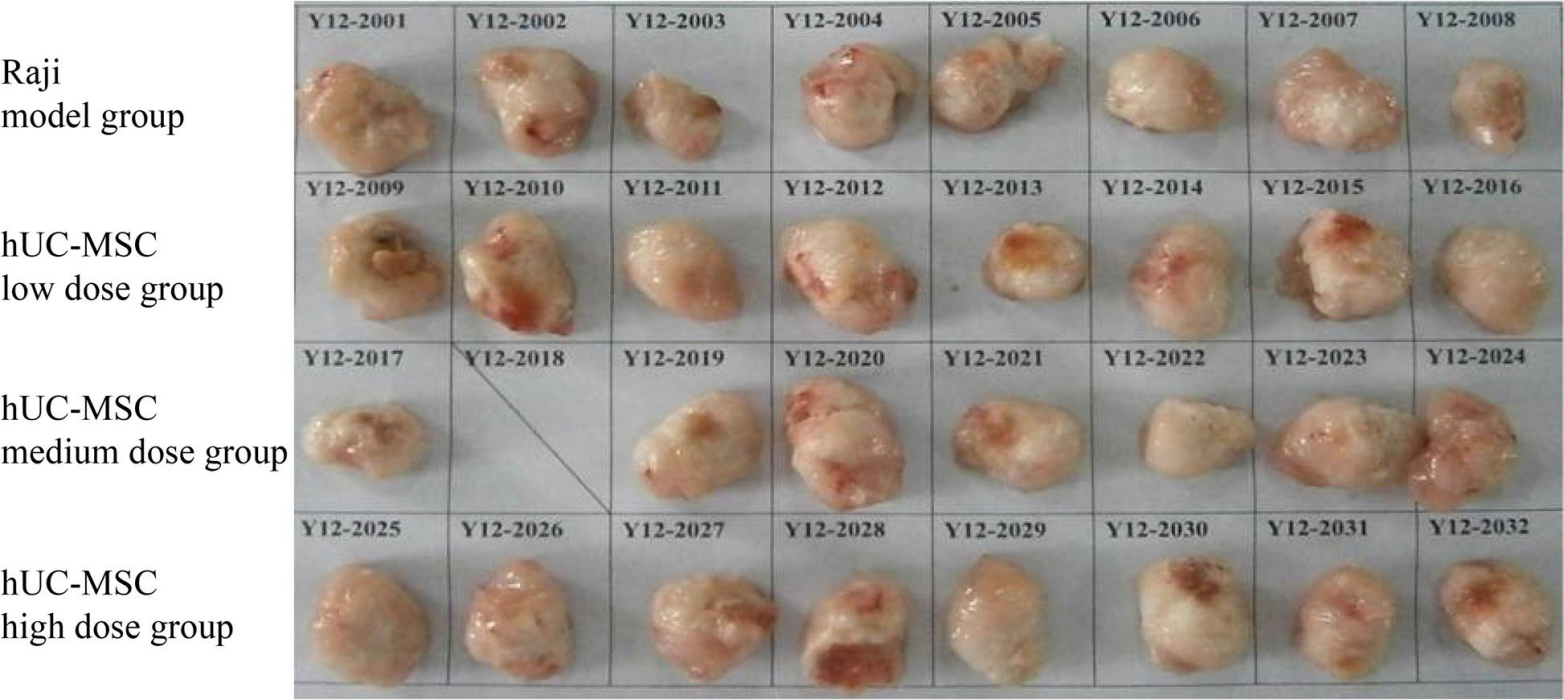
Photos of tumors from each group on day 37 (Raji). The tumors in each group were the same size, and any perceived differences were not significant. This result indicated that low, medium and high doses of hUC-MSCs did not significantly promote or inhibit the growth and metastasis of Raji cell-derived tumors

**Fig. 8:**
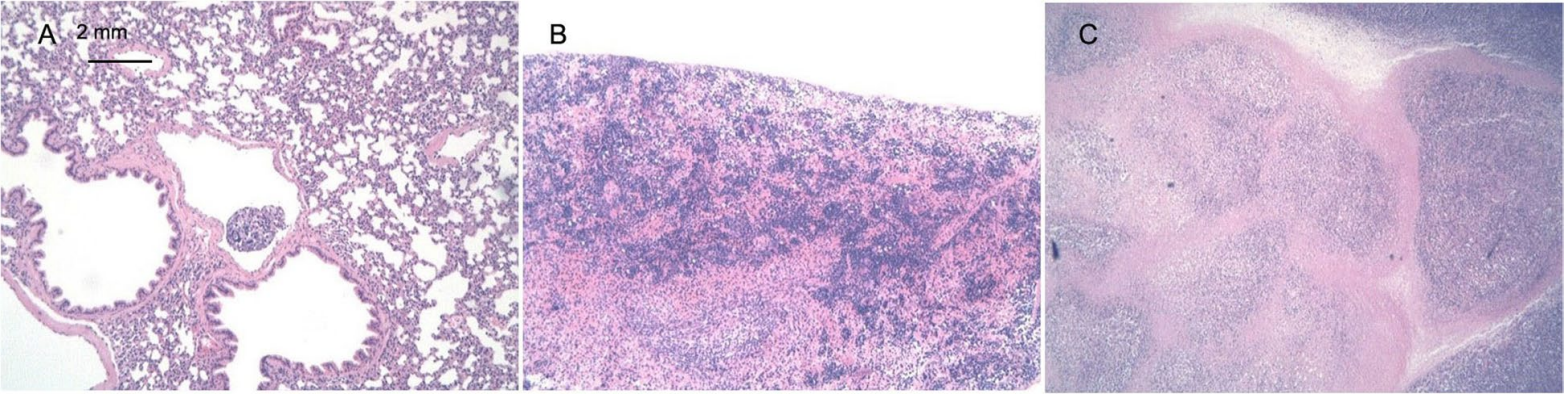
Histopathological examination of tissues and Raji cell-derived tumors in NOD SCID mice administered hUC-MSCs (HE staining, 10 ×). **A** Raji cells metastasized through small veins in the lungs of the animals in the model group. **B** There were more extramedullary hematopoietic cell colonies in the spleens of the animals in the low-dose group. **C** The axillary tissue of animals in the medium-dose group exhibited central necrosis in the inoculated tumor cell mass

The general anatomical and pathological results are shown in Figs. 7 and 8.

### The tumorigenesis potential of MSCs administered via tail vein injection

The mice injected with MSCs via their tail vein were euthanized, and gross anatomical analyses and HE staining of each organ were performed. The general structure and pathological features of each organ were normal, which proved that the MSCs were not tumorigenic.

## Discussion

With the great clinical potential of MSCs, preclinical safety assessments of these cells have become important. Due to the biological characteristics of different tissue sources and processing methods of MSCs, there are certain safety risks for clinical research and basic experiments. Some studies have suggested that in vitro culture of bone marrow-derived MSCs for longer times, allowing increasing passage numbers, leads to increased tumorigenic ability [2]. In the process of long-term culture and passage, the nucleus-to-cytoplasm ratio and telomerase activity of bone marrow MSCs increased, and there was a tendency of spontaneous malignant transformation. When the transformed cells were transplanted into NOD SCID mice, tumor growth was observed [5]. It has been reported that interleukin-6 secreted by MSCs can increase the secretion of endothelin 1 by cancer cells, thereby enhancing tumor growth and angiogenesis [6], while other reports have reached the opposite conclusion. MSCs can significantly inhibit melanoma cell growth and reduce tumor blood vessel density. It is believed that MSCs are suitable for inhibiting angiogenesis during tumor treatment [7]. Secchiero et al. [8] preliminarily created a lymphoma model in NOD SCID mice and found that transplanting MSCs slowed the occurrence and development of tumors, prolonged mouse survival, and exerted anticancer effects. The different immunoregulatory activities and secretion abilities of MSCs from different sources may determine whether MSCs are tumorigenic or exert different effects on tumors. Therefore, in vivo research on the tumorigenic and tumor-promoting abilities of MSCs is the most important part of quality assessments before clinical application.

The NOD SCID mouse selected in this study has the scid gene deletion, which causes severe combined immunodeficiency, on the NOD background (spontaneously develops type I diabetes and a variety of autoimmune diseases). NOD SCID mice have a single recessive mutation in a gene on chromosome 16 and abnormal VDJ recombinase activity, which can prevent the differentiation of functional T and B lymphocytes, resulting in defects in both cellular and humoral immune functions. This model is not used for xenografts because immune rejection occurs, but it is suitable for research on the tumorigenicity of MSCs. Almost all tumors, including hematological tumors, can grow in SCID mice. The tumor transplantation success rate is high, tumors form quickly, and tumor progression occurs rapidly. All tumor-bearing mice can be observed in a short period of time for changes in the disease course. Therefore, this model is also the animal model of choice for studying tumor promotion by MSCs.

In the tumorigenesis experiment, the negative control was the MRC-5 cell line, which are normal, nontumorigenic human embryonic lung fibroblasts; the small size of these cells enables easy absorption after subcutaneous injection. The nodules formed after the inoculation of these cells were smaller, and no nodules remained during the 16-week observation period, indicating proper establishment of this negative control. The positive control was the HeLa human cervical cancer cell line, which is a tumor cell line with obvious tumorigenicity. All 10 animals in this group had nodules, and nodule volume showed continuous growth. These nodules were pathologically confirmed to be tumorous, indicating appropriate establishment of the positive control. To comprehensively study the tumorigenicity of different generations of MSCs, three passages of hUC-MSCs from two sources were subcutaneously inoculated into mice, and most of the animals in each group exhibited fibrotic proliferative nodules. Pathological examination of tissue sections showed that these nodules were nonneoplastic. A reason for this finding may be that after hUC-MSCs were inoculated subcutaneously in mice, the large cell volume made it difficult for the body to completely absorb or clear the cells, thereby stimulating the infiltration of inflammatory cells, such as monocytes, into the injection site and the surrounding tissues. Connective tissue hyperplasia occurs upon a robust and extensive inflammatory response; when the body cannot remove necrotic tissue, a large area of ​​necrosis forms in the center of the nodule, and the necrotic tissue stimulates the proliferation of the surrounding fibrous tissue to encapsulate the necrosis and limit its development. Therefore, the animals inoculated with hUC-MSCs in this experiment did not appear to have obvious nodules within 4 days after inoculation, and as the tissue was repaired, the necrotic tissue was removed, and the nodules gradually decreased in size until they disappeared. In this experiment, the trends in nodule formation and disappearance after injection were the same for the three different passages of hUC-MSCs from two sources, indicating no significant difference in the tumorigenicity of the cells in each group.

In the tumor promotion experiment, the tumor nodules in each HeLa group grew steadily, indicating that this subcutaneous tumor model was successfully established in NOD SCID mice. During the experiment, tumor rupture occurred in some animals in each group, but the incidence was similar across the groups and did not cause large fluctuations in the data. Therefore, it is believed that tumor rupture in this experiment had no effect on the evaluation of the results. During the experiment, one animal died in each of the high-dose and medium-dose hUC-MSC groups, and there were no obvious gross anatomical abnormalities. However, the sudden death of an animal in the medium-dose group was not accompanied by abnormal changes in organs, so the animal was considered to have died in a manner not related to the injection of hUC-MSCs. Therefore, it is believed that the injection of hUC-MSCs does not promote the metastasis of tumor cells.

In the Raji transplant tumor model experiment, the tumor nodules in each group grew steadily, indicating the successful establishment of the human B cell lymphoma Raji cell subcutaneous transplant model in the NOD SCID mice. Some animals began to develop tumor ulceration on D29, and the incidence was high. Due to the large tumor diameter and frequent tumor rupture, the experiment ended early on D37 (data collected through D36), but this did not affect the evaluation of the results. During the experiment, 1 animal died after injection of each dose of hUC-MSCs, and no obvious gross anatomical abnormalities were observed. The pathological examination showed conditions such as extensive lung tissue hemorrhage and focal renal infarction. Therefore, this death was suspected to be the result of spontaneous disease that was unrelated to the injection of hUC-MSCs. Except for tumor cell masses in the pulmonary venules of 1 animal in the model group, no cancer cell metastasis was observed in the main organs of the animals in the other groups. However, due to the short experimental period, it could not be determined whether hUC-MSCs promote or inhibit the metastasis of tumor cells.

UC-MSCs have great clinical therapeutic potential, which has been supported by various studies. At present, mesenchymal stem cell-based treatments have been marketed worldwide, but before clinical use, safety is a major issue. Although there have been studies on the tumor-promoting and antitumor properties of mesenchymal stem cells, these studies have not systematically investigated mesenchymal stem cells for a preclinical use or as an emerging drug candidate to evaluate their tumorigenesis and tumor-promoting properties. This study was conducted with an UC-MSC product to comprehensively evaluate its tumorigenicity and tumor-promoting effects. In terms of tumorigenicity, three generations of low, medium, and high passage numbers from different donors are selected. Three doses of (low, medium and high) were used to evaluate the tumor-promoting effects, basically covering the various possibilities of clinical use. This is the most comprehensive evaluation of the safety of mesenchymal stem cell therapy thus far. Second, tumorigenicity assessment is the most important step in preclinical safety assessment, and this should be one of the indicators that must be evaluated in mesenchymal stem cell products on the market. In many pretreatment safety evaluations of mesenchymal stem cells, to quickly determine the results, tumorigenicity evaluation is based on in vitro experiments, such as telomerase assays and soft clone agar formation experiments. This study is based on in vivo animal experiments, and the results are more reliable. The above summarizes the innovations of this research: 1) This research was designed from the perspective of cell-based drugs, which makes it more appropriate for addressing the current problems related to the translation of UC-MSCs into clinical use; 2) The selection of cell sources, generations and dosages were comprehensive and systematic, and the results are referential; 3) Tumorigenic results in animals are more reliable than tumorigenic results in vitro.

## Conclusions

In summary, under the experimental conditions, three generations (P5, P7, and P10) of UC-MSCs derived from two donors (hUC-MSC1 and hUC-MSC2) were subcutaneously inoculated at a dose of 1 × 10^7^ cells into NOD SCID mice. No tumorous nodules grew under the skin at the inoculation site of these mice. In the solid tumor model (human cervical cancer HeLa cells) and the hematological model (human lymphoma Raji cells), NOD SCID mice that were subcutaneously inoculated with cancer cells showed no increase or decrease in tumor growth or metastasis upon treatment with hUC-MSCs. However, the interaction between transplanted UC-MSCs and the host immune system is complicated; large randomized clinical trials are needed to ascertain whether this interaction eventually causes cancer and promotes cancer proliferation and metastasis.

## Supplementary Information

**Additional file 1.** **Supplementary materials :** Figure S1 Antibody-labeled cells were shown to have high expression of CD105(95.4%), CD73(99.2%), and CD90(96%) and low or no expression of CD45(0.14%) and CD34(0.16%) by flow cytometry. These results met the criteria for identifying mesenchymal stem cells by surface marker expression.  Table S1 The tumorigenicity test results of NOD/SCID mice administered human umbilical cord mesenchymal stem cell injection (body weight (g, mean±SD)). Table S2 The tumorigenicity test results of NOD/SCID mice administered human umbilical cord mesenchymal stem cell injection(nodule volume (mm3, mean±SD)). Table S3 The effect of human umbilical cord mesenchymal stem cell injection on tumor growth in HeLa cell-derived tumor-bearing NOD/SCID mice (weight (g, mean±SD)). Table S4 The effect of human umbilical cord mesenchymal stem cell injection on tumor growth in HeLa cell-derived tumor-bearing NOD/SCID mice (tumor volume (mm3, mean±SD)). Table S5 The effect of human umbilical cord mesenchymal stem cell injection on tumor growth in HeLa cell-derived tumor-bearing NOD/SCID mice (RTV). Table S6 The effect of human umbilical cord mesenchymal stem cell injection on tumor growth in HeLa cell-derived tumor-bearing NOD/SCID mice (T/C%). Table S7 The effect of human umbilical cord mesenchymal stem cell injection on tumor growth in HeLa cell-derived tumor-bearing NOD/SCID mice (tumor weight (g, mean±SD) and tumor weight inhibition rate (%)). Table S8 The effect of human umbilical cord mesenchymal stem cell injection on tumor growth in Raji cell-derived tumor-bearing NOD/SCID mice (weight (g, mean±SD)). Table S9: Effects of human umbilical cord mesenchymal stem cell injection on tumor growth in Raji cell-derived tumor-bearing NOD/SCID mice (tumor volume (mm3, mean±SD)). Table S10 The effect of human umbilical cord mesenchymal stem cell injection on tumor growth in Raji cell-derived tumor-bearing NOD/SCID mice (RTV). Table S11 The effect of human umbilical cord mesenchymal stem cell injection on tumor growth in Raji cell-derived tumor-bearing NOD/SCID mice (T/C%). Table S12 The effect of human umbilical cord mesenchymal stem cell injection on tumor growth in Raji cell-derived tumor-bearing NOD/SCID mice (tumor weight (g, mean±SD) and tumor weight inhibition rate (%)). 

## Data Availability

All the data generated or analyzed during this study are included in this published article [and its supplementary information files].

## Abbreviation

UC-MSCs: Umbilical cord mesenchymal stem cells
MSCs: mesenchymal stem cells
TERT: telomerase reverse transcriptase gene
MRC-5: human embryonic lung fibroblasts
HeLa: human cervical cancer cells
Raji: human B cell lymphoma cells
RTV: relative tumor volume
