# Supplementary material for: Lack of tumorigenesis and protumorigenic activity of human umbilical cord mesenchymal stem cells in NOD SCID mice
Source: BMC Cancer. 2022 Mar 22;22:307. doi: 10.1186/s12885-022-09431-5 (PMC8941803; doi:10.1186/s12885-022-09431-5)
Supplement: Supplementary file 1 — Additional file 1. Supplementary materials : Figure S1 Antibody-labeled cells were shown to have high expression of CD105(95.4%), CD73(99.2%), and CD90(96%) and low or no expression of CD45(0.14%) and CD34(0.16%) by flow cytometry. These results met the criteria for identifying mesenchymal stem cells by surface marker expression. Table S1 The tumorigenicity test results of NOD/SCID mice administered human umbilical cord mesenchymal stem cell injection (body weight (g, mean±SD)). Table S2 The tumorigenicity test results of NOD/SCID mice administered human umbilical cord mesenchymal stem cell injection(nodule volume (mm3, mean±SD)). Table S3 The effect of human umbilical cord mesenchymal stem cell injection on tumor growth in HeLa cell-derived tumor-bearing NOD/SCID mice (weight (g, mean±SD)). Table S4 The effect of human umbilical cord mesenchymal stem cell injection on tumor growth in HeLa cell-derived tumor-bearing NOD/SCID mice (tumor volume (mm3, mean±SD)). Table S5 The effect of human umbilical cord mesenchymal stem cell injection on tumor growth in HeLa cell-derived tumor-bearing NOD/SCID mice (RTV). Table S6 The effect of human umbilical cord mesenchymal stem cell injection on tumor growth in HeLa cell-derived tumor-bearing NOD/SCID mice (T/C%). Table S7 The effect of human umbilical cord mesenchymal stem cell injection on tumor growth in HeLa cell-derived tumor-bearing NOD/SCID mice (tumor weight (g, mean±SD) and tumor weight inhibition rate (%)). Table S8 The effect of human umbilical cord mesenchymal stem cell injection on tumor growth in Raji cell-derived tumor-bearing NOD/SCID mice (weight (g, mean±SD)). Table S9: Effects of human umbilical cord mesenchymal stem cell injection on tumor growth in Raji cell-derived tumor-bearing NOD/SCID mice (tumor volume (mm3, mean±SD)). Table S10 The effect of human umbilical cord mesenchymal stem cell injection on tumor growth in Raji cell-derived tumor-bearing NOD/SCID mice (RTV). Table S11 The effect of human umbilica [file 12885_2022_9431_MOESM1_ESM.doc]

Figure S1


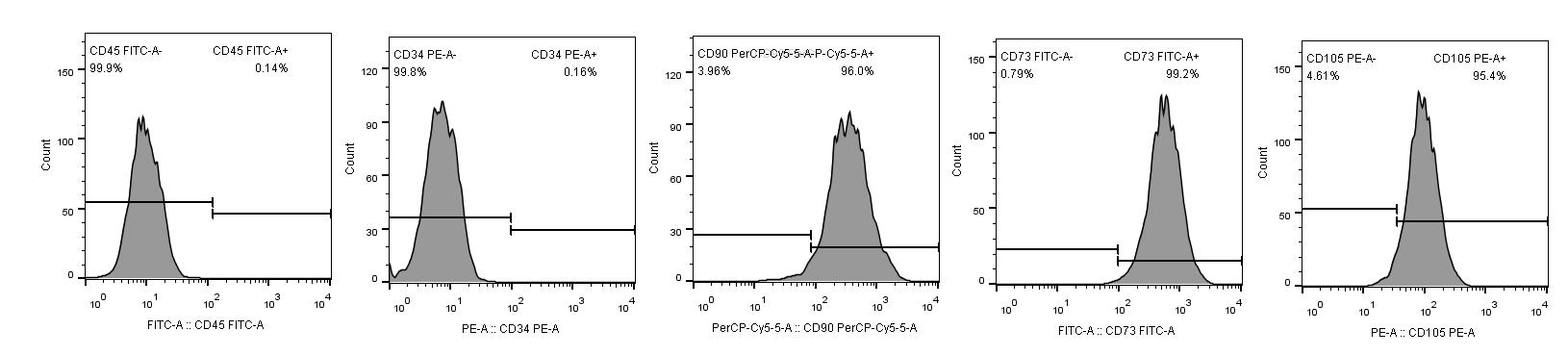


Figure S1 Antibody-labeled cells were shown to have high expression of CD105(95.4%), CD73(99.2%), and CD90(96%) and low or no expression of CD45(0.14%) and CD34(0.16%) by flow cytometry. These results met the criteria for identifying mesenchymal stem cells by surface marker expression.

Table S1 The tumorigenicity test results of NOD/SCID mice administered human umbilical cord mesenchymal stem cell injection (body weight (g, mean±SD))

| Group | Cell name/lot number | Seed cells  (Pcs/only) | Number of animals  (*n*) | D1 | D3 | D6 | D10 | D13 | D17 | D20 |
| --- | --- | --- | --- | --- | --- | --- | --- | --- | --- | --- |
| 1 | MRC-5 | 1×10^7^ | 10 | 20.4±1.6 | 20.6±2.0 | 20.7±1.9 | 21.0±1.8 | 21.6±1.8 | 21.9±2.1 | 22.6±1.8 |
| 2 | HeLa | 1×10^6^ | 10 | 20.8±1.4 | 20.7±1.3 | 20.5±1.9 | 20.9±1.6 | 21.5±1.8 | 21.9±1.7 | 21.9±1.9 |
| 3 | hUC-MSC1-P5 | 1×10^7^ | 10 | 20.5±1.3 | 20.1±1.4 | 20.5±1.2 | 21.1±1.2 | 20.7±2.5 | 22.6±1.2 | 22.8±1.7 |
| 4 | hUC-MSC1-P7 | 1×10^7^ | 10 | 21.2±1.8 | 20.8±1.8 | 21.4±1.6 | 21.6±1.8 | 22.8±1.5 | 23.7±1.6 | 24.2±1.8 |
| 5 | hUC-MSC1-P10 | 1×10^7^ | 10 | 21.0±1.8 | 20.6±1.8 | 21.4±2.4 | 21.7±2.2 | 22.5±2.7 | 22.5±2.7 | 22.9±2.7 |
| 6 | hUC-MSC2-P5 | 1×10^7^ | 10 | 20.4±1.6 | 20.0±1.6 | 19.5±1.4 | 20.1±1.5 | 20.4±1.4 | 20.9±2.3 | 21.1±2.6 |
| 7 | hUC-MSC2-P7 | 1×10^7^ | 10 | 20.9±1.7 | 20.5±1.7 | 20.4±1.8 | 20.7±1.9 | 21.2±1.7 | 21.8±2.0 | 22.0±2.7 |
| 8 | hUC-MSC2-P10 | 1×10^7^ | 10 | 20.6±0.9 | 20.0±1.4 | 20.1±1.7 | 20.6±1.4 | 21.7±1.2 | 22.4±1.5 | 23.0±1.8 |

Table S1 (Continued) The tumorigenicity test results of NOD/SCID mice administered human umbilical cord mesenchymal stem cell injection(body weight (g, ))

| Group | Cell name/lot number | Seed cells  (Pcs/only) | Number of animals (*n*) | D24 | D27 | D31 | D34 | D38 | D41 | D45 |
| --- | --- | --- | --- | --- | --- | --- | --- | --- | --- | --- |
| 1 | MRC-5 | 1×10^7^ | 5 | 22.4±2.2 | 22.1±3.4 | 23.5±3.1 | 23.2±3.6 | 23.9±3.1 | 23.9±2.6 | 23.9±2.7 |
| 2 | HeLa | 1×10^6^ | 10 | 22.3±1.8 | 22.0±2.7 | 23.0±2.0 | 23.3±2.1 | 23.9±1.8 | 23.9±1.9 | 24.0±1.8 |
| 3 | hUC-MSC1-P5 | 1×10^7^ | 5 | 23.9±2.1 | 24.1±1.7 | 24.5±1.5 | 24.1±1.4 | 24.3±1.7 | 24.8±1.7 | 24.5±1.8 |
| 4 | hUC-MSC1-P7 | 1×10^7^ | 5 | 25.1±1.7 | 25.2±1.9 | 25.8±2.2 | 25.7±2.5 | 26.1±2.3 | 26.2±2.0 | 26.5±2.0 |
| 5 | hUC-MSC1-P10 | 1×10^7^ | 5 | 23.7±2.8 | 23.9±2.9 | 24.7±2.7 | 25.2±2.6 | 25.8±2.5 | 26.2±1.9 | 26.0±1.8 |
| 6 | hUC-MSC2-P5 | 1×10^7^ | 5 | 21.7±2.7 | 21.9±2.2 | 22.8±1.9 | 21.9±1.4 | 22.5±1.9 | 23.2±2.1 | 23.2±2.5 |
| 7 | hUC-MSC2-P7 | 1×10^7^ | 5 | 23.2±3.1 | 23.6±3.3 | 24.0±3.1 | 23.8±3.2 | 24.5±2.8 | 23.9±1.9 | 23.5±1.4 |
| 8 | hUC-MSC2-P10 | 1×10^7^ | 5 | 23.9±1.9 | 24.0±1.3 | 24.6±1.3 | 24.9±0.6 | 25.1±0.9 | 25.0±1.0 | 25.1±0.8 |

Table S1 (Continued) The tumorigenicity test results of NOD/SCID mice administered human umbilical cord mesenchymal stem cell injection(body weight (g, ))

| Group | Cell name/lot number | Seed cells  (Pcs/only) | Number of animals  (*n*) | D48 | D52 | D55 | D59 | D62 | D66 | D69 |
| --- | --- | --- | --- | --- | --- | --- | --- | --- | --- | --- |
| 1 | MRC-5 | 1×10^7^ | 5 | 24.5±2.8 | 24.3±2.6 | 24.2±3.2 | 24.4±2.9 | 24.4±2.6 | 25.1±3.5 | 25.3±3.2 |
| 2 | HeLa | 1×10^6^ | 10 | 24.2±1.9 | 24.6±1.8 | 24.4±1.7 | 24.4±1.6 | 0.0±0.0 | 0.0±0.0 | 0.0±0.0 |
| 3 | hUC-MSC1-P5 | 1×10^7^ | 5 | 24.5±1.9 | 24.9±1.5 | 25.1±2.1 | 25.3±2.1 | 25.1±1.7 | 25.8±1.7 | 25.9±1.8 |
| 4 | hUC-MSC1-P7 | 1×10^7^ | 5 | 26.7±2.1 | 27.1±2.1 | 27.1±2.3 | 27.0±2.4 | 27.2±2.2 | 27.2±2.6 | 27.7±2.5 |
| 5 | hUC-MSC1-P10 | 1×10^7^ | 5 | 26.8±2.5 | 27.0±2.2 | 26.2±2.4 | 26.7±2.1 | 27.3±2.3 | 27.6±2.6 | 27.4±1.9 |
| 6 | hUC-MSC2-P5 | 1×10^7^ | 5 | 23.5±2.4 | 24.2±2.2 | 23.7±2.5 | 23.5±2.8 | 23.9±2.6 | 23.8±2.4 | 23.6±2.1 |
| 7 | hUC-MSC2-P7 | 1×10^7^ | 5 | 23.2±0.7 | 22.9±1.1 | 22.6±1.2 | 22.7±1.1 | 23.1±0.8 | 23.5±1.4 | 23.2±0.9 |
| 8 | hUC-MSC2-P10 | 1×10^7^ | 5 | 24.7±0.6 | 25.2±1.4 | 24.7±1.2 | 24.3±1.3 | 24.9±1.2 | 24.9±1.6 | 25.6±1.4 |

Table S1 (Continued) The tumorigenicity test results of NOD/SCID mice administered human umbilical cord mesenchymal stem cell injection(body weight (g, ))

| Group | Cell name/lot number | Seed cells  (Pcs/only) | Number of animals  (*n*) | D73 | D76 | D80 | D83 | D87 | D90 |
| --- | --- | --- | --- | --- | --- | --- | --- | --- | --- |
| 1 | MRC-5 | 1×10^7^ | 5 | 25.4±3.2 | 25.3±3.8 | 25.8±3.5 | 25.6±3.1 | 25.6±3.3 | 25.5±3.5 |
| 2 | HeLa | 1×10^6^ | 10 | 0.0±0.0 | 0.0±0.0 | 0.0±0.0 | 0.0±0.0 | 0.0±0.0 | 0.0±0.0 |
| 3 | hUC-MSC1-P5 | 1×10^7^ | 5 | 26.0±2.1 | 25.8±1.9 | 26.1±2.1 | 26.2±2.0 | 25.6±2.1 | 25.1±2.3 |
| 4 | hUC-MSC1-P7 | 1×10^7^ | 5 | 28.4±2.3 | 28.0±2.7 | 28.1±2.8 | 28.2±3.2 | 28.5±2.8 | 27.8±2.9 |
| 5 | hUC-MSC1-P10 | 1×10^7^ | 5 | 27.6±2.3 | 27.6±2.8 | 28.5±2.9 | 27.8±2.6 | 27.9±2.7 | 27.7±2.5 |
| 6 | hUC-MSC2-P5 | 1×10^7^ | 5 | 24.2±2.6 | 24.1±2.6 | 24.1±2.9 | 23.9±3.1 | 24.0±3.3 | 24.0±2.9 |
| 7 | hUC-MSC2-P7 | 1×10^7^ | 5 | 23.5±0.8 | 23.2±1.1 | 23.5±1.3 | 23.5±0.8 | 23.6±1.1 | 23.7±1.0 |
| 8 | hUC-MSC2-P10 | 1×10^7^ | 5 | 25.5±1.4 | 25.6±2.0 | 26.2±1.0 | 26.3±1.4 | 26.3±1.0 | 25.8±0.5 |

Table S1 (Continued) The tumorigenicity test results of NOD/SCID mice administered human umbilical cord mesenchymal stem cell injection(body weight (g, ))

| Group | Cell name/lot number | Seed cells  (Pcs/only) | Number of animals  (*n*) | D97 | D101 | D104 | D108 | D111 | D113 |
| --- | --- | --- | --- | --- | --- | --- | --- | --- | --- |
| 1 | MRC-5 | 1×10^7^ | 5 | 26.1±3.7 | 26.0±3.4 | 25.7±3.9 | 25.8±3.5 | 25.9±3.5 | 23.6±3.0 |
| 2 | HeLa | 1×10^6^ | 10 | 0.0±0.0 | 0.0±0.0 | 0.0±0.0 | 0.0±0.0 | 0.0±0.0 | 0.0±0.0 |
| 3 | hUC-MSC1-P5 | 1×10^7^ | 5 | 25.5±2.8 | 25.4±2.7 | 25.8±3.1 | 25.8±2.7 | 26.3±3.2 | 23.6±2.8 |
| 4 | hUC-MSC1-P7 | 1×10^7^ | 5 | 28.3±2.8 | 28.4±2.2 | 29.0±2.7 | 28.1±2.5 | 27.7±3.1 | 25.9±2.0 |
| 5 | hUC-MSC1-P10 | 1×10^7^ | 5 | 28.2±3.0 | 28.3±2.7 | 28.4±2.9 | 28.6±2.5 | 28.1±2.2 | 25.5±2.4 |
| 6 | hUC-MSC2-P5 | 1×10^7^ | 5 | 24.2±2.8 | 24.0±2.9 | 24.0±3.1 | 24.1±2.7 | 24.0±2.6 | 21.3±2.9 |
| 7 | hUC-MSC2-P7 | 1×10^7^ | 5 | 23.8±1.5 | 24.0±1.4 | 24.4±1.0 | 24.1±0.9 | 24.0±0.6 | 21.7±0.4 |
| 8 | hUC-MSC2-P10 | 1×10^7^ | 5 | 26.0±1.4 | 26.1±1.1 | 26.6±1.3 | 25.9±1.2 | 25.3±1.4 | 23.7±0.8 |

Table S2 The tumorigenicity test results of NOD/SCID mice administered human umbilical cord mesenchymal stem cell injection(nodule volume (mm^3^, mean±SD))

| Group | Cell name/lot number | Seed cells  (Pcs/only) | Number of animals  (*n*) | D5 | D6 | D7 | D8 | D9 |
| --- | --- | --- | --- | --- | --- | --- | --- | --- |
| 1 | MRC-5 | 1×10^7^ | 10 | 0.000±0.000 | 0.000±0.000 | 0.000±0.000 | 0.000±0.000 | 0.000±0.000 |
| 2 | HeLa | 1×10^6^ | 10 | 0.000±0.000 | 0.000±0.000 | 0.000±0.000 | 18.021±11.035 | 31.474±11.780 |
| 3 | hUC-MSC1-P5 | 1×10^7^ | 10 | 364.194±100.884 | 341.144±126.822 | 305.903±133.229 | 213.924±86.902 | 152.252±55.917 |
| 4 | hUC-MSC1-P7 | 1×10^7^ | 10 | 335.597±51.144 | 294.736±75.431 | 286.923±124.026 | 157.167±40.234 | 142.124±35.911 |
| 5 | hUC-MSC1-P10 | 1×10^7^ | 10 | 291.968±118.826 | 261.044±110.447 | 214.430±91.587 | 143.641±62.077 | 81.217±45.280 |
| 6 | hUC-MSC2-P5 | 1×10^7^ | 10 | 348.279±166.113 | 274.454±164.097 | 300.025±167.361 | 203.617±106.630 | 196.534±99.633 |
| 7 | hUC-MSC2-P7 | 1×10^7^ | 10 | 320.437±161.283 | 232.690±92.476 | 218.710±77.089 | 202.677±78.765 | 122.243±53.171 |
| 8 | hUC-MSC2-P10 | 1×10^7^ | 10 | 291.830±143.748 | 252.312±86.235 | 228.377±70.897 | 157.888±42.456 | 132.632±52.939 |

Note: nodule data were not statistically analyzed

Table S2 (Continued) The tumorigenicity test results of NOD/SCID mice administered human umbilical cord mesenchymal stem cell injection (nodule volume (mm^3^, mean±SD))

| Group | Cell name/lot number | Seed cells  (Pcs/only) | Number of animals  (*n*) | D10 | D11 | D12 | D13 | D14 |
| --- | --- | --- | --- | --- | --- | --- | --- | --- |
| 1 | MRC-5 | 1×10^7^ | 10 | 0.000±0.000 | 0.000±0.000 | 0.000±0.000 | 0.000±0.000 | 0.000±0.000 |
| 2 | HeLa | 1×10^6^ | 10 | 48.075±21.456 | 59.978±22.807 | 80.112±27.500 | 113.576±41.339 | 140.544±39.418 |
| 3 | hUC-MSC1-P5 | 1×10^7^ | 10 | 102.735±36.492 | 75.952±35.450 | 29.090±32.178 | 21.207±26.650 | 2.465±5.243 |
| 4 | hUC-MSC1-P7 | 1×10^7^ | 10 | 81.211±25.225 | 66.044±28.345 | 32.008±36.546 | 21.695±25.774 | 4.860±9.750 |
| 5 | hUC-MSC1-P10 | 1×10^7^ | 10 | 71.396±40.082 | 54.806±30.391 | 24.186±35.727 | 16.930±29.087 | 3.273±6.929 |
| 6 | hUC-MSC2-P5 | 1×10^7^ | 10 | 121.832±62.579 | 73.715±42.623 | 24.003±29.833 | 14.896±19.140 | 1.091±3.451 |
| 7 | hUC-MSC2-P7 | 1×10^7^ | 10 | 81.328±22.671 | 63.819±23.900 | 27.222±31.660 | 18.754±23.557 | 1.676±3.806 |
| 8 | hUC-MSC2-P10 | 1×10^7^ | 10 | 66.726±25.110 | 47.648±26.251 | 27.263±34.964 | 14.506±17.010 | 2.417±3.995 |

Table S2 (Continued) The tumorigenicity test results of NOD/SCID mice administered human umbilical cord mesenchymal stem cell injection (nodule volume (mm^3^, ))

| Group | Cell name/lot number | Seed cells  (Pcs/only) | Number of animals  (*n*) | D17 | D20 | D24 | D27 | D34 |
| --- | --- | --- | --- | --- | --- | --- | --- | --- |
| 1 | MRC-5 | 1×10^7^ | 5 | 0.000±0.000 | 0.000±0.000 | 0.000±0.000 | 0.000±0.000 | 0.000±0.000 |
| 2 | HeLa | 1×10^6^ | 10 | 174.159±48.755 | 194.489±51.561 | 258.643±74.111 | 362.979±91.268 | 545.960±176.368 |
| 3 | hUC-MSC1-P5 | 1×10^7^ | 5 | 0.000±0.000 | 0.000±0.000 | 0.000±0.000 | 0.000±0.000 | 0.000±0.000 |
| 4 | hUC-MSC1-P7 | 1×10^7^ | 5 | 0.000±0.000 | 0.000±0.000 | 0.000±0.000 | 0.000±0.000 | 0.000±0.000 |
| 5 | hUC-MSC1-P10 | 1×10^7^ | 5 | 0.000±0.000 | 0.000±0.000 | 0.000±0.000 | 0.000±0.000 | 0.000±0.000 |
| 6 | hUC-MSC2-P5 | 1×10^7^ | 5 | 0.000±0.000 | 0.000±0.000 | 0.000±0.000 | 0.000±0.000 | 0.000±0.000 |
| 7 | hUC-MSC2-P7 | 1×10^7^ | 5 | 0.000±0.000 | 0.000±0.000 | 0.000±0.000 | 0.000±0.000 | 0.000±0.000 |
| 8 | hUC-MSC2-P10 | 1×10^7^ | 5 | 0.000±0.000 | 0.000±0.000 | 0.000±0.000 | 0.000±0.000 | 0.000±0.000 |

Table S2 (Continued) The tumorigenicity test results of NOD/SCID mice administered human umbilical cord mesenchymal stem cell injection (nodule volume (mm^3^, mean±SD))

| Group | Cell name/lot number | Seed cells  (Pcs/only) | Number of animals  (*n*) | D41 | D48 | D55 | D62 | D69 |
| --- | --- | --- | --- | --- | --- | --- | --- | --- |
| 1 | MRC-5 | 1×10^7^ | 5 | 0.000±0.000 | 0.000±0.000 | 0.000±0.000 | 0.000±0.000 | 0.000±0.000 |
| 2 | HeLa | 1×10^6^ | 10 | 1036.692±301.456 | 1155.541±375.357 | 2031.105±451.815 | 0.000±0.000 | 0.000±0.000 |
| 3 | hUC-MSC1-P5 | 1×10^7^ | 5 | 0.000±0.000 | 0.000±0.000 | 0.000±0.000 | 0.000±0.000 | 0.000±0.000 |
| 4 | hUC-MSC1-P7 | 1×10^7^ | 5 | 0.000±0.000 | 0.000±0.000 | 0.000±0.000 | 0.000±0.000 | 0.000±0.000 |
| 5 | hUC-MSC1-P10 | 1×10^7^ | 5 | 0.000±0.000 | 0.000±0.000 | 0.000±0.000 | 0.000±0.000 | 0.000±0.000 |
| 6 | hUC-MSC2-P5 | 1×10^7^ | 5 | 0.000±0.000 | 0.000±0.000 | 0.000±0.000 | 0.000±0.000 | 0.000±0.000 |
| 7 | hUC-MSC2-P7 | 1×10^7^ | 5 | 0.000±0.000 | 0.000±0.000 | 0.000±0.000 | 0.000±0.000 | 0.000±0.000 |
| 8 | hUC-MSC2-P10 | 1×10^7^ | 5 | 0.000±0.000 | 0.000±0.000 | 0.000±0.000 | 0.000±0.000 | 0.000±0.000 |

Note: All the remaining nodules in animals disappeared from D76 to D113, so they are not listed in the table.

Table S3 The effect of human umbilical cord mesenchymal stem cell injection on tumor growth in HeLa cell-derived tumor-bearing NOD/SCID mice (weight (g, ))

| Group | Dose (mg/kg) | Number of animals  (n) | D1 | D4 | D8 | D11 | D15 | D18 | D22 | D25 | D29 |
| --- | --- | --- | --- | --- | --- | --- | --- | --- | --- | --- | --- |
| Model group | Cell dilution | 8 | 18.1±0.9 | 18.4±1.0 | 19.0±1.1 | 19.0±1.0 | 19.4±1.2 | 19.5±1.1 | 19.8±1.0 | 19.8±1.4 | 20.1±1.5 |
| hUC-MSC low-dose group | 1×10^7^ | 8 | 18.2±1.2 | 15.7±2.8 | 18.6±1.6 | 18.8±1.7 | 18.6±1.7 | 18.9±1.7 | 19.2±1.9 | 19.4±1.8 | 19.4±2.0 |
| hUC-MSC medium-dose group | 2×10^7^ | 8 | 18.2±1.4 | 18.2±1.2 | 18.4±1.1 | 18.7±1.3 | 18.7±1.2 | 18.8±1.2 | 19.2±1.1 | 19.5±1.2 | 18.5±1.6 |
| hUC-MSC high-dose group | 4×10^7^ | 8 | 17.9±1.0 | 18.3±1.2 | 18.4±1.1 | 18.6±1.3 | 17.5±2.1 | 19.0±1.5 | 19.6±1.4 | 19.9±1.7 | 20.4±1.7 |

Table S3 (Continued) Effect of human umbilical cord mesenchymal stem cell injection on tumor growth in HeLa cell-derived tumor-bearing NOD/SCID mice (weight (g, mean±SD))

| Group | Dose (mg/kg) | Number of animals  (n) | D32 | D36 | D39 | D43 | D46 | D50 | D53 | D57 |
| --- | --- | --- | --- | --- | --- | --- | --- | --- | --- | --- |
| Model group | Cell dilution | 8 | 20.0±1.4 | 20.4±1.7 | 20.5±1.6 | 20.5±1.8 | 19.9±2.0 | 21.0±1.9 | 20.8±1.9 | 21.3±2.2 |
| hUC-MSC low-dose group | 1×10^7^ | 8 | 19.6±2.4 | 19.5±2.6 | 19.5±2.5 | 19.3±2.9 | 20.2±2.5 | 20.6±2.5 | 20.6±2.2 | 20.7±2.2 |
| hUC-MSC medium dose group | 2×10^7^ | 8 | 19.7±1.7 | 19.8±1.6 | 19.8±1.4 | 19.9±1.6 | 19.8±1.8 | 20.0±1.9 | 20.1±1.8 | 20.1±1.6 |
| hUC-MSC high-dose group | 4×10^7^ | 8 | 20.6±1.7 | 20.6±2.0 | 20.9±2.0 | 20.8±2.0 | 21.1±2.0 | 21.1±2.0 | 21.1±2.2 | 21.2±2.4 |

Note: 1. hUC-MSC low-dose group, n=7 from D46;

2. hUC-MSC medium-dose group, n=7 starting from D32.

Table S4 The effect of human umbilical cord mesenchymal stem cell injection on tumor growth in HeLa cell-derived tumor-bearing NOD/SCID mice (tumor volume (mm^3^, mean±SD))

| Group | Dose (mg/kg) | Number of animals  (n) | D1 | D4 | D8 | D11 | D15 | D18 |
| --- | --- | --- | --- | --- | --- | --- | --- | --- |
| Model group | Cell dilution | 8 | 75.86±16.77 | 109.51±26.84 | 168.10±37.92 | 244.31±55.84 | 336.94±60.77 | 418.62±82.76 |
| hUC-MSC low-dose group | 1×10^7^ | 8 | 75.38±25.00 | 117.11±24.88 | 163.83±53.05 | 207.83±37.66 | 304.76±63.73 | 385.11±79.47 |
| hUC-MSC medium dose group | 2×10^7^ | 8 | 74.02±14.93 | 106.92±26.47 | 161.80±34.81 | 178.29±42.16 | 267.51±49.20 | 334.42±45.41 |
| hUC-MSC high-dose group | 4×10^7^ | 8 | 74.89±17.37 | 124.04±33.60 | 203.80±51.74 | 218.52±52.61 | 308.92±61.27 | 393.26±108.84 |

Table S4 (Continued) The effect of human umbilical cord mesenchymal stem cell injection on tumor growth in HeLa cell-derived tumor-bearing NOD/SCID mice (tumor volume (mm^3^, ))

| Group | Dose (mg/kg) | Number of animals  (n) | D22 | D25 | D29 | D32 | D36 | D39 |
| --- | --- | --- | --- | --- | --- | --- | --- | --- |
| Model group | Cell dilution | 8 | 583.96±139.05 | 695.68±209.03 | 833.83±229.45 | 954.78±262.25 | 1125.32±276.16 | 1307.74±217.87 |
| hUC-MSC low-dose group | 1×10^7^ | 8 | 514.39±91.19 | 616.53±192.68 | 705.95±129.41 | 802.61±134.07 | 1032.61±242.42 | 1164.41±258.54 |
| hUC-MSC medium dose group | 2×10^7^ | 8 | 419.29±64.95 | 512.43±107.60 | 622.11±140.76 | 739.10±132.77 | 841.96±179.31 | 1048.59±244.05 |
| hUC-MSC high-dose group | 4×10^7^ | 8 | 543.85±121.64 | 653.06±184.60 | 805.74±212.60 | 908.41±220.14 | 1005.55±237.14 | 1322.45±313.68 |

Note: hUC-MSC medium-dose group, n=7 starting from D32.

Table S4 (Continued) The effect of human umbilical cord mesenchymal stem cell injection on tumor growth in HeLa cell-derived tumor-bearing NOD/SCID mice (tumor volume (mm^3^, ))

| Group | Dose (mg/kg) | Number of animals  (n) | D43 | D46 | D50 | D53 | D57 |
| --- | --- | --- | --- | --- | --- | --- | --- |
| Model group | Cell dilution | 8 | 2135.90±608.81 | 2161.86±630.90 | 2741.70±443.54 | 2802.50±486.49 | 3028.58±710.55 |
| hUC-MSC low-dose group | 1×10^7^ | 8 | 1772.98±489.14 | 1859.26±326.86 | 2589.76±447.09 | 2504.06±367.09 | 2839.68±436.18 |
| hUC-MSC medium dose group | 2×10^7^ | 8 | 1575.73±452.71 | 1771.28±572.84 | 2101.66±720.32 | 2226.29±652.09 | 2316.22±532.66 |
| hUC-MSC high-dose group | 4×10^7^ | 8 | 1899.50±528.86 | 2134.07±533.20 | 2579.05±658.04 | 2619.30±742.28 | 2625.85±616.23 |

Note: 1. hUC-MSC low-dose group, n=7 from D46;

2. hUC-MSC medium-dose group, n=7 starting from D32.

Table S5 The effect of human umbilical cord mesenchymal stem cell injection on tumor growth in HeLa cell-derived tumor-bearing NOD/SCID mice (RTV)

| Group | Dose (mg/kg) | Number of animals  (n) | D4 | D8 | D11 | D15 | D18 | D22 |
| --- | --- | --- | --- | --- | --- | --- | --- | --- |
| Model group | Cell dilution | 8 | 1.49±0.45 | 2.30±0.74 | 3.26±0.61 | 4.51±0.70 | 5.60±1.00 | 7.77±1.47 |
| hUC-MSC low-dose group | 1×10^7^ | 8 | 1.65±0.44 | 2.24±0.55 | 2.97±0.88 | 4.38±1.44 | 5.59±1.89 | 7.36±2.08 |
| hUC-MSC medium-dose group | 2×10^7^ | 8 | 1.46±0.33 | 2.21±0.40 | 2.43±0.46 | 3.68±0.71 | 4.64±0.94 | 5.83±1.38 |
| hUC-MSC high-dose group | 4×10^7^ | 8 | 1.67±0.31 | 2.77±0.66 | 2.94±0.52 | 4.18±0.59 | 5.24±1.17 | 7.33±1.30 |

Table S5 (Continued) Effect of human umbilical cord mesenchymal stem cell injection on tumor growth in HeLa cell-derived tumor-bearing NOD/SCID mice (RTV)

| Group | Dose (mg/kg) | Number of animals  (n) | D25 | D29 | D32 | D36 | D39 |
| --- | --- | --- | --- | --- | --- | --- | --- |
| Model group | Cell dilution | 8 | 9.08±1.35 | 10.97±1.54 | 12.59±2.12 | 14.85±2.23 | 17.50±2.27 |
| hUC-MSC low-dose group | 1×10^7^ | 8 | 8.82±3.18 | 10.30±3.71 | 11.85±4.69 | 14.97±5.29 | 17.14±6.88 |
| hUC-MSC medium-dose group | 2×10^7^ | 8 | 7.19±2.19 | 8.72±2.76 | 10.19±2.86 | 11.55±3.33 | 14.38±4.06 |
| hUC-MSC high-dose group | 4×10^7^ | 8 | 8.81±2.23 | 11.05±3.30 | 12.33±3.02 | 13.60±2.99 | 17.82±3.65 |

Note: hUC-MSC medium-dose group, n=7 starting from D32.

Table S5 (Continued) Effect of human umbilical cord mesenchymal stem cell injection on tumor growth in HeLa cell-derived tumor-bearing NOD/SCID mice (RTV)

| Group | Dose (mg/kg) | Number of animals  (n) | D43 | D46 | D50 | D53 | D57 |
| --- | --- | --- | --- | --- | --- | --- | --- |
| Model group | Cell dilution | 8 | 28.09±4.88 | 28.36±4.91 | 36.69±4.76 | 37.40±4.35 | 40.26±7.01 |
| hUC-MSC low-dose group | 1×10^7^ | 8 | 26.34±11.59 | 28.17±10.51 | 39.55±15.02 | 37.92±13.38 | 43.17±15.82 |
| hUC-MSC medium-dose group | 2×10^7^ | 8 | 21.30±6.01 | 24.04±8.12 | 28.54±9.81 | 30.36±9.66 | 31.44±7.47 |
| hUC-MSC high-dose group | 4×10^7^ | 8 | 25.94±7.59 | 29.18±8.22 | 35.21±9.37 | 35.86±11.06 | 35.99±9.38 |

Note: 1. hUC-MSC low-dose group, n=7 from D46;

2. hUC-MSC medium-dose group, n=7 starting from D32.

Table S6 The effect of human umbilical cord mesenchymal stem cell injection on tumor growth in HeLa cell-derived tumor-bearing NOD/SCID mice (T/C%)

| Group | Dose (mg/kg) | Number of animals  (n) | D4 | D8 | D11 | D15 | D18 | D22 | D25 | D29 |
| --- | --- | --- | --- | --- | --- | --- | --- | --- | --- | --- |
| hUC-MSC low-dose group | 1×10^7^ | 8 | 110.69 | 97.30 | 91.12 | 97.27 | 99.88 | 94.78 | 97.13 | 93.89 |
| hUC-MSC medium-dose group | 2×10^7^ | 8 | 97.99 | 96.09 | 74.69 | 81.78 | 82.81 | 75.02 | 79.22 | 79.40 |
| hUC-MSC high-dose group | 4×10^7^ | 8 | 112.31 | 120.30 | 90.31 | 92.76 | 93.64 | 94.34 | 97.01 | 100.68 |

Table S6 (Continued) Effect of human umbilical cord mesenchymal stem cell injection on tumor growth in HeLa cell-derived tumor-bearing NOD/SCID mice (T/C%)

| Group | Dose (mg/kg) | Number of animals  (n) | D32 | D36 | D39 | D43 | D46 | D50 | D53 | D57 |
| --- | --- | --- | --- | --- | --- | --- | --- | --- | --- | --- |
| hUC-MSC low-dose group | 1×10^7^ | 8 | 94.17 | 100.85 | 97.93 | 93.78 | 99.32 | 107.79 | 101.39 | 107.23 |
| hUC-MSC medium-dose group | 2×10^7^ | 8 | 80.94 | 77.82 | 82.15 | 75.85 | 84.74 | 77.79 | 81.18 | 78.11 |
| hUC-MSC high-dose group | 4×10^7^ | 8 | 98.00 | 91.63 | 101.79 | 92.34 | 102.86 | 95.96 | 95.89 | 89.39 |

Note: 1. hUC-MSC low-dose group, n=7 from D46;

2. hUC-MSC medium-dose group, n=7 starting from D32.

Table S7 The effect of human umbilical cord mesenchymal stem cell injection on tumor growth in HeLa cell-derived tumor-bearing NOD/SCID mice (tumor weight (g, mean±SD) and tumor weight inhibition rate (%))

| Group | Dose (mg/kg) | Number of animals  (n) | D57 | Tumor weight inhibition rate（%） |
| --- | --- | --- | --- | --- |
| Model group | Cell dilution | 8 | 2.455±0.555 | — |
| hUC-MSC low-dose group | 1×10^7^ | 7 | 2.349±0.357 | 4.30 |
| hUC-MSC medium-dose group | 2×10^7^ | 7 | 2.082±0.690 | 15.19 |
| hUC-MSC high-dose group | 4×10^7^ | 8 | 2.491±0.734 | -1.45 |

Table S8 The effect of human umbilical cord mesenchymal stem cell injection on tumor growth in Raji cell-derived tumor-bearing NOD/SCID mice (weight (g, ))

| Group | Dose (mg/kg) | Number of animals  (n) | D1 | D4 | D8 | D11 | D15 | D18 |
| --- | --- | --- | --- | --- | --- | --- | --- | --- |
| Model group | Cell dilution | 8 | 18.2±1.3 | 18.3±1.9 | 18.7±1.7 | 17.4±2.0 | 19.3±1.7 | 19.6±1.6 |
| hUC-MSC low-dose group | 1×10^7^ | 8 | 18.0±1.3 | 18.3±1.3 | 19.0±1.3 | 19.3±1.2 | 19.8±1.4 | 20.3±1.4 |
| hUC-MSC medium-dose group | 2×10^7^ | 8 | 18.1±0.8 | 18.1±1.1 | 17.4±1.5 | 18.8±1.6 | 17.9±1.8 | 19.4±1.3 |
| hUC-MSC high-dose group | 4×10^7^ | 8 | 18.3±1.2 | 19.0±1.4 | 18.3±1.7 | 19.5±1.6 | 20.1±1.3 | 20.4±1.7 |

Table S8 (Continued) Effect of human umbilical cord mesenchymal stem cell injection on tumor growth in Raji cell-derived tumor-bearing NOD/SCID mice (weight (g, mean±SD))

| Group | Dose (mg/kg) | Number of animals  (n) | D22 | D25 | D29 | D32 | D36 |
| --- | --- | --- | --- | --- | --- | --- | --- |
| Model group | Cell dilution | 8 | 20.1±1.6 | 19.9±1.7 | 20.5±1.9 | 20.7±2.1 | 20.5±2.7 |
| hUC-MSC low-dose group | 1×10^7^ | 8 | 21.1±1.4 | 21.1±1.6 | 21.7±1.5 | 21.8±1.7 | 21.2±2.3 |
| hUC-MSC medium-dose group | 2×10^7^ | 8 | 20.2±1.4 | 20.6±1.5 | 21.4±1.3 | 21.6±1.9 | 21.5±3.0 |
| hUC-MSC high-dose group | 4×10^7^ | 8 | 21.2±1.4 | 21.6±1.2 | 22.6±1.4 | 22.9±1.2 | 22.7±1.6 |

Note: hUC-MSC medium-dose group, D36 n=7.

Table S9: Effects of human umbilical cord mesenchymal stem cell injection on tumor growth in Raji cell-derived tumor-bearing NOD/SCID mice (tumor volume (mm^3^,))

| Group | Dose (mg/kg) | Number of animals  (n) | D1 | D4 | D8 | D11 | D15 | D18 |
| --- | --- | --- | --- | --- | --- | --- | --- | --- |
| Model group | Cell dilution | 8 | 65.10±22.21 | 86.38±25.56 | 148.81±39.39 | 239.02±87.94 | 389.51±111.87 | 583.31±275.06 |
| hUC-MSC low-dose group | 1×10^7^ | 8 | 65.98±23.71 | 127.01±39.94 | 191.66±65.13 | 348.97±122.83 | 593.88±198.07 | 718.63±227.48 |
| hUC-MSC medium-dose group | 2×10^7^ | 8 | 64.43±20.64 | 95.10±35.16 | 152.03±44.04 | 314.01±118.79 | 473.20±243.79 | 604.44±272.24 |
| hUC-MSC high-dose group | 4×10^7^ | 8 | 65.29±16.19 | 107.98±46.05 | 197.43±133.89 | 341.96±176.61 | 510.65±225.04 | 677.29±330.75 |

Table S9 (Continued) Effect of human umbilical cord mesenchymal stem cell injection on tumor growth in Raji cell-derived tumor-bearing NOD/SCID mice (tumor volume (mm^3^, ))

| Group | Dose (mg/kg) | Number of animals  (n) | D22 | D25 | D29 | D32 | D36 |
| --- | --- | --- | --- | --- | --- | --- | --- |
| Model group | Cell dilution | 8 | 712.27±244.14 | 1160.49±413.31 | 1508.48±484.88 | 1800.45±541.62 | 2474.87±738.57 |
| hUC-MSC low-dose group | 1×10^7^ | 8 | 1121.11±345.08 | 1584.36±395.22 | 2182.67±674.79 | 2647.93±685.34* | 2878.42±567.72 |
| hUC-MSC medium-dose group | 2×10^7^ | 8 | 895.51±332.64 | 1286.27±413.97 | 1830.77±491.79 | 2445.54±598.13 | 2813.67±663.98 |
| hUC-MSC high-dose group | 4×10^7^ | 8 | 1112.71±440.32 | 1513.32±452.32 | 2063.18±544.24 | 2679.76±574.68* | 2990.84±231.94 |

Note: 1. hUC-MSC medium-dose group, D36 n=7;

2. Compared with the model group, * means P≤0.05.

Table S10 The effect of human umbilical cord mesenchymal stem cell injection on tumor growth in Raji cell-derived tumor-bearing NOD/SCID mice (RTV)

| Group | Dose (mg/kg) | Number of animals  (n) | D4 | D8 | D11 | D15 | D18 |
| --- | --- | --- | --- | --- | --- | --- | --- |
| Model group | Cell dilution | 8 | 1.37±0.24 | 2.36±0.42 | 3.80±1.29 | 6.35±1.96 | 9.42±4.39 |
| hUC-MSC low-dose group | 1×10^7^ | 8 | 1.96±0.29** | 3.00±0.67 | 5.57±2.03 | 9.24±2.06 | 11.54±3.98 |
| hUC-MSC medium-dose group | 2×10^7^ | 8 | 1.49±0.26 | 2.43±0.46 | 5.00±1.19 | 7.18±2.02 | 9.33±2.30 |
| hUC-MSC high-dose group | 4×10^7^ | 8 | 1.64±0.53 | 2.99±1.75 | 5.32±2.53 | 8.07±3.55 | 10.66±5.23 |

Note: Compared with the model group, ** means P≤0.01.

Table S10 (Continued) The effect of human umbilical cord mesenchymal stem cell injection on tumor growth in Raji cell-derived tumor-bearing NOD/SCID mice (RTV)

| Group | Dose (mg/kg) | Number of animals  (n) | D22 | D25 | D29 | D32 | D36 |
| --- | --- | --- | --- | --- | --- | --- | --- |
| Model group | Cell dilution | 8 | 11.73±4.70 | 18.97±7.57 | 25.28±10.75 | 30.06±11.87 | 41.68±18.20 |
| hUC-MSC low-dose group | 1×10^7^ | 8 | 17.89±5.84 | 25.86±8.83 | 34.84±11.65 | 43.20±15.04 | 47.69±15.55 |
| hUC-MSC medium-dose group | 2×10^7^ | 8 | 14.58±4.83 | 21.69±9.16 | 31.71±15.14 | 43.29±22.74 | 53.60±29.79 |
| hUC-MSC high-dose group | 4×10^7^ | 8 | 17.68±7.53 | 24.34±8.90 | 32.94±10.29 | 42.78±11.67 | 48.26±11.95 |

Note: hUC-MSC medium-dose group, D36 n=7.

Table S11 The effect of human umbilical cord mesenchymal stem cell injection on tumor growth in Raji cell-derived tumor-bearing NOD/SCID mice (T/C%)

| Group | Dose (mg/kg) | Number of animals  (n) | D4 | D8 | D11 | D15 | D18 | D22 | D25 | D29 | D32 | D36 |
| --- | --- | --- | --- | --- | --- | --- | --- | --- | --- | --- | --- | --- |
| hUC-MSC low-dose group | 1×10^7^ | 8 | 143.89 | 127.31 | 146.62 | 145.51 | 122.49 | 152.46 | 136.28 | 137.84 | 143.70 | 114.42 |
| hUC-MSC medium-dose group | 2×10^7^ | 8 | 108.78 | 103.09 | 131.68 | 113.12 | 99.06 | 124.29 | 114.30 | 125.46 | 144.01 | 128.60 |
| hUC-MSC high-dose group | 4×10^7^ | 8 | 120.27 | 126.85 | 140.07 | 127.16 | 113.13 | 150.68 | 128.30 | 130.31 | 142.31 | 115.79 |

Note: hUC-MSC medium-dose group, D36 n=7.

Table S12 The effect of human umbilical cord mesenchymal stem cell injection on tumor growth in Raji cell-derived tumor-bearing NOD/SCID mice (tumor weight (g, mean±SD) and tumor weight inhibition rate (%))

| Group | Dose (mg/kg) | Number of animals  (n) | D36 | Tumor weight inhibition rate（%） |
| --- | --- | --- | --- | --- |
| Model group | Cell dilution | 8 | 2.214±0.872 | — |
| hUC-MSC low-dose group | 1×10^7^ | 8 | 2.509±0.607 | -13.34 |
| hUC-MSC medium-dose group | 2×10^7^ | 7 | 2.607±0.900 | -17.76 |
| hUC-MSC high-dose group | 4×10^7^ | 8 | 2.796±0.275 | -26.31 |
